# A New Method for Rapid Screening of End-Point PCR Products: Application to Single Genome Amplified HIV and SIV Envelope Amplicons

**DOI:** 10.1371/journal.pone.0128188

**Published:** 2015-06-08

**Authors:** Laurent Houzet, Claire Deleage, Anne-Pascale Satie, Laetitia Merlande, Dominique Mahe, Nathalie Dejucq-Rainsford

**Affiliations:** Inserm U 1085-IRSET, Université de Rennes 1, Structure Fédérative Recherche Biosit, Campus de Beaulieu, Rennes, France; University of Pittsburgh Center for Vaccine Research, UNITED STATES

## Abstract

PCR is the most widely applied technique for large scale screening of bacterial clones, mouse genotypes, virus genomes etc. A drawback of large PCR screening is that amplicon analysis is usually performed using gel electrophoresis, a step that is very labor intensive, tedious and chemical waste generating. Single genome amplification (SGA) is used to characterize the diversity and evolutionary dynamics of virus populations within infected hosts. SGA is based on the isolation of single template molecule using limiting dilution followed by nested PCR amplification and requires the analysis of hundreds of reactions per sample, making large scale SGA studies very challenging. Here we present a novel approach entitled Long Amplicon Melt Profiling (LAMP) based on the analysis of the melting profile of the PCR reactions using SYBR Green and/or EvaGreen fluorescent dyes. The LAMP method represents an attractive alternative to gel electrophoresis and enables the quick discrimination of positive reactions. We validate LAMP for SIV and HIV env-SGA, in 96- and 384-well plate formats. Because the melt profiling allows the screening of several thousands of PCR reactions in a cost-effective, rapid and robust way, we believe it will greatly facilitate any large scale PCR screening.

## Introduction

Analysis of SIV/HIV quasispecies genetic diversity is used to assess virus compartmentalization between fluids and organs in infected individuals and to detect drug resistant viruses. Cloning and sequencing of bulk PCR products has been progressively abandoned because of the Taq polymerase induced mutations and recombinations, and of the non-proportional representation of individual sequences. While the development of next generation sequencing (NGS) methods allows a faster and deeper analysis of viral quasispecies, this approach remains limited by the existence of bulk PCR-induced viral recombinations, the restricted length of the fragment to analyze, and the need for extensive bioinformatics analyses. In this context, Single genome amplification (SGA) has emerged as the state of the art method and is currently the most commonly used approach for the genetic analysis of HIV and SIV strain populations. SGA is based on the amplification of a single template molecule isolated using limiting dilution method [1–4]. In essence, viral template is diluted and PCR is performed using multiple replicates to identify a dose where no more than 30% of reactions are PCR positive. With this limited amount of template, the positive reactions have been mathematically determined to originate from a single genome more than 80% of the time. Amplicons are then submitted to direct sequencing and the potentially remaining reactions with evidence of priming from more than one original template excluded based on inspection of the sequence for mixed bases. HIV and SIV envelope gene (env) sequences are the most widely used for phylogenetic analysis as they are highly variable and therefore very useful for discriminating viral variants. In addition, full length SIV/HIV env amplicons provide important information on virus fitness, tropism and immune selection pressure [5–10]. Advantages of SGA method over other approaches (e.g. bulk PCR or NGS) include enhanced specificity for the detection of rare individuals, compatibility with short and long amplicons, absence of bulk PCR induced artefacts (e.g. recombination and nucleotide misincorporations), and no requirement for extensive bioinformatics analysis. However, this methodology has two drawbacks: 1) the high number of PCR reaction replicates to be performed; 2) the requirement for a very labor intensive and time consuming gel electrophoresis screening step to identify the positive reactions amongst the numerous negative reactions. Thus, between one and two hundred PCR reactions are generally necessary to harvest the 24 amplicons required for a robust phylogenetic analysis, as no more than 30% of the PCR reactions have to be positive to ensure single clone amplification [4]. Although the development of absolute quantification by qPCR of viral cDNAs in samples facilitated the determination of the optimal working dilution, reducing this number from an average of 245 reactions to 135 reactions [11], the need for extensive gel electrophoresis analyses of PCR reaction products remains an important issue in large scale studies involving numerous samples, or in SGA core facilities, as it is labor intensive, tedious and generates lots of chemical waste.

Here we present a new strategy to quickly and inexpensively determine the positivity and specificity of the numerous PCR reactions required for HIV and SIV env SGA assay, which bypasses the labor-intensive and time consuming electrophoresis gel analysis step. The method we devised and called Long Amplicon Melt Profiling (LAMP) is based on our finding that unspliced full env HIV/SIV amplicon exhibits a specific melting profile using SYBR Green or EvaGreen dyes. This enables the quick discrimination of full length env amplicons against spliced RNA, unspecific products and negative PCR reactions. We describe the protocols we developed and validated for the rapid screening of HIV and SIV amplicons in different matrixes (blood plasma, semen, tissues and isolated cells) and their implementation for large scale studies, using only a small fraction of the SGA reaction product.

## Materials and Methods

### Ethics statement

Adult cynomolgus macaques (Macaca fascicularis) imported from Mauritius were housed in the facilities of the ‘‘Commissariat à l’Energie Atomique et aux Energies Alternatives” (CEA, Fontenay-aux-Roses, France). Non-human primates (NHP) are used at the CEA in accordance with French national regulation and under national veterinary inspectors (CEA Permit Number A 92-032-02). The CEA is in compliance with Standards for Human Care and Use of Laboratory of the Office for Laboratory Animal Welfare (OLAW, USA) under OLAW Assurance number #A5826-01. The use of NHP at CEA is in accordance with the recommendation of the newly published European Directive (2010/63, recommendation Nu9). Animals were housed in adjoining individual cages allowing social interactions, under controlled conditions of humidity, temperature and light (12-hour light/12-hour dark cycles). Water was available *ad libitum*. Animals were monitored and fed 1–2 times daily with commercial monkey chow and fruits by trained personnel. Macaques were provided with environmental enrichment including toys, novel foodstuffs and music under the supervision of the CEA Animal Welfare Body. The protocols employed were approved under statement number 10–060 (November 13^th^ 2012) by the ethical committee of the CEA “Comité d’Ethique en Expérimentation Animale” registered by the French Research Ministry under number 44. The animals were used under the supervision of the veterinarians in charge of the animal facility. Experimental procedures were conducted after animal sedation with ketamine chlorydrate (Rhone-Merieux, Lyon, France, 10 mg/kg). Tissues from the MGT were collected during animal necropsy at the end of the treatment after sedation of animals (ketamine chlorhydrate 10 mk/kg) followed by euthanasia (sodium pentobarbital 180 mg/kg). It should be stressed that none of the animal were specifically used for this work, since semen, blood, lymph nodes and male genital organs were collected in the course of other studies ([12, 13]) thus no suffering was specifically associated with the procedure to collect these samples. This approach is fully in accordance with the 3R and reduces the number of animal used as recommended by the Directive 2010/63/CE (article 18 sharing tissues and organs).

### Study animals and sample collection

Frozen (-80°C) blood plasma, semen cells and plasma, lymph node and male genital tissue samples collected from SIVmac251-infected adult cynomolgus macaques (Macaca fascicularis) in the context of other studies ([12, 13]) were used.

### Cell culture and HIV infection

Jurkat cell line was cultured in RPMI medium with 10% fetal calf serum (FCS) and 2 mM L-glutamine. For HIV-1 infection, 18 x 10^6^ cells were incubated with 300 μl of a stock of HIV-IIIb (corresponding to 500 ng p24) overnight and then rinsed three times with PBS. Cells were harvested 8 days post-infection and cell pellets frozen for RNA extraction.

### Viral RNA extraction and cDNA synthesis

RNA was extracted from semen and blood plasma and infected cells using Trizol-LS and Trizol Reagent respectively according to manufacturer’s instructions (Life tech). Epididymis and inguinal lymph node tissue samples were homogenized with a TissueLyser II (QIAGEN) and total RNA was isolated using Trizol Reagent (Life tech) according to manufacturers’ protocols. Reverse transcription of RNA to cDNA was performed as described previously [1, 3] with minor modifications. Briefly, RNA, deoxynucleoside triphosphates (1mM) and 1 μM primer EnvR1 (5’-TGTAATAAATCCCTTCCAGTCCCCCC-3’) for SIV and HIVenv3out (5’-TTGCTACTTGTGATTGCTCCATGT-3’) for HIV were incubated for 6 min at 65°C followed by incubation at 4°C for 3 min. Then a mix of the following was added: 4 μl of 5x First-Strand buffer, 1 μl of 100mM Dithiothreitol, 0.5 μl of RNaseOUT recombinant Ribonuclease inhibitor (40 units/μl; Invitrogen Life Technologies) and 1 μl of SuperScript III Reverse Transcriptase (200 units/μl; Invitrogen Life Technologies). The reaction mixture was then incubated at 53°C for 60 min followed by 15 min at 70°C. cDNA was stored at -80°C until further analysis.

### SIV and HIV single-genome nested amplification (SGA)

Full-length 3.2-kb SIV and HIV env gene amplicons were obtained as described previously [1–3] with minor modifications. For SIV SGA, optimal SGA working dilution was determined for each cDNA sample using 4-fold serial dilutions (ranging from 1/4 to 1/256 for all samples except for inguinal lymph node tissue sample, 1/4 to 1/4096) in double distilled water distributed in replicates of 6 PCR reactions. According to Poisson distribution, the DNA dilution yielding positive PCR products in no more than 30% of the wells will provide amplicons resulting from single molecule amplification in over 80% of these wells. The corresponding dilution, designed as SGA working dilution, was used to perform additional PCR amplifications. SGA PCR conditions were as follows: the first-round PCR was performed using 1 μl cDNA dilution in 96 well plate with 1x Phusion HF Buffer, 0.2 mM of each deoxynucleoside triphosphate, 0.3 μM of primers SIVsm/macEnvF1 (5’-CCTCCCCCTCCAGGACTAGC-3’) and SIVsm/macEnvR1 (5’- TGTAATAAATCCCTTCCAGTCCCCCC- 3’), and 0.016 U/μl Phusion Hot Start Flex DNA polymerase (New England Biolabs) in a 25 μl reaction volume. The following cycling conditions were used: 98°C for 45 s followed by 35 cycles of 98°C for 15 s, 55°C for 30 s, 72°C for 6 min and a final extension of 72°C for 10 min. The second-round PCR reaction was carried out using 1 μl of the first-round product with the primers SIVmacEnvF2 (5’-TATAATAGACATGGAGACACCCTTGAGGGAGC- 3’) and SIVsmEnvR2 (5’-ATGAGACATRTCTATTGCCAATTTGTA- 3’) and the same PCR mixture than in the first-round. The cycling conditions were: 98°C for 45 s followed by 40 cycles of 98°C for 15 s, 55°C for 30 s, 72°C for 5 min and a final extension of 72°C for 10 min. Amplicons were inspected on 1.2% agarose gel and/or using SYBR/Eva-Green melting analysis. Because HIV SGA was performed using ex-vivo infected cell samples containing high amount of viral RNA, we used QPCR to determine HIV cDNA concentration in RT reaction samples and get a first approximation of the SGA working dilution. QPCR was performed with 0.2 μl RT reaction using the sense sHIV-1306 (5'-TCAGCATTATCAGAAGGAGCCACC-3’) and antisens aHIV-1541 (5'-TCATCCATCCTATTTGTTCCTGAAG-3’) primers with IQ SYBR Green supermix in a 7500 real Time PCR System instrument (Applied Biosystems). A standard curve ranging from 10^2^ to 10^6^ copies of pNL4.3 plasmid was generated for absolute quantification. The cDNA concentration value was used to calculate an approximate SGA working dilution corresponding to 0.3 copies/μl. 7 successive 4-fold serial dilutions centered on this value were performed in distilled water containing 10 ng/μl tRNA (Sigma-Aldrich) and distributed in replicates of 6 PCR reactions to determine the exact SGA working dilution to use. The same PCR conditions as for SIV were used with the following primers: HIVenv5out (5’-TAGAGCCCTGGAAGCATCCAGGAAG-3’) and HIVenv3out (5’-TTGCTACTTGTGATTGCTCCATGT-3’) for the first-round PCR and HIVenv5in (5’-TTAGGCATCTCCTATGGCAGGAAGAAG-3’) and HIVenv3in (5’-GTCTCGAGATACTGCTCCCACCC-3’) for the second round.

### Melt profiling

Unless otherwise specified, the melting analysis was carried out in 96-well plate with 1.2 μl of second-round PCR reaction product, 2 μl of IQ SYBR Green supermix or Precision Melt Supermix containing EvaGreen Dye (Bio-Rad) and 0.16 μl of ROX Passive Reference Dye (Bio-Rad) in a 8 μl reaction mixture using a 7500 real Time PCR System instrument (Applied Biosystems). The following melt curve run method was used: 95°C for 15 s, 60°C for 1 min followed by an increase to 95°C with a ramp rate of 1% and continuous data acquisition (40 min long). When specified, melt profiling was performed using either CFX96 Touch or CFX384 Touch Real time PCR detection system instruments (Bio-Rad) with the same melting reaction mixture as given above except that no ROX Passive Reference Dye was added. Melting run method was as follows: 95°C for 15 s, 60°C for 1 min followed by an increase to 95°C with an increment of 0.5°C and reading after 2 s (25 and 35 min long for 96 and 384-well instruments respectively). Melting profile was analyzed to isolate the positive reactions with the onboard software using the methods described in the text.

## Results

### SYBR Green melting analysis of the 3.2-kb SIV env SGA-amplicon

**Fig 1:**
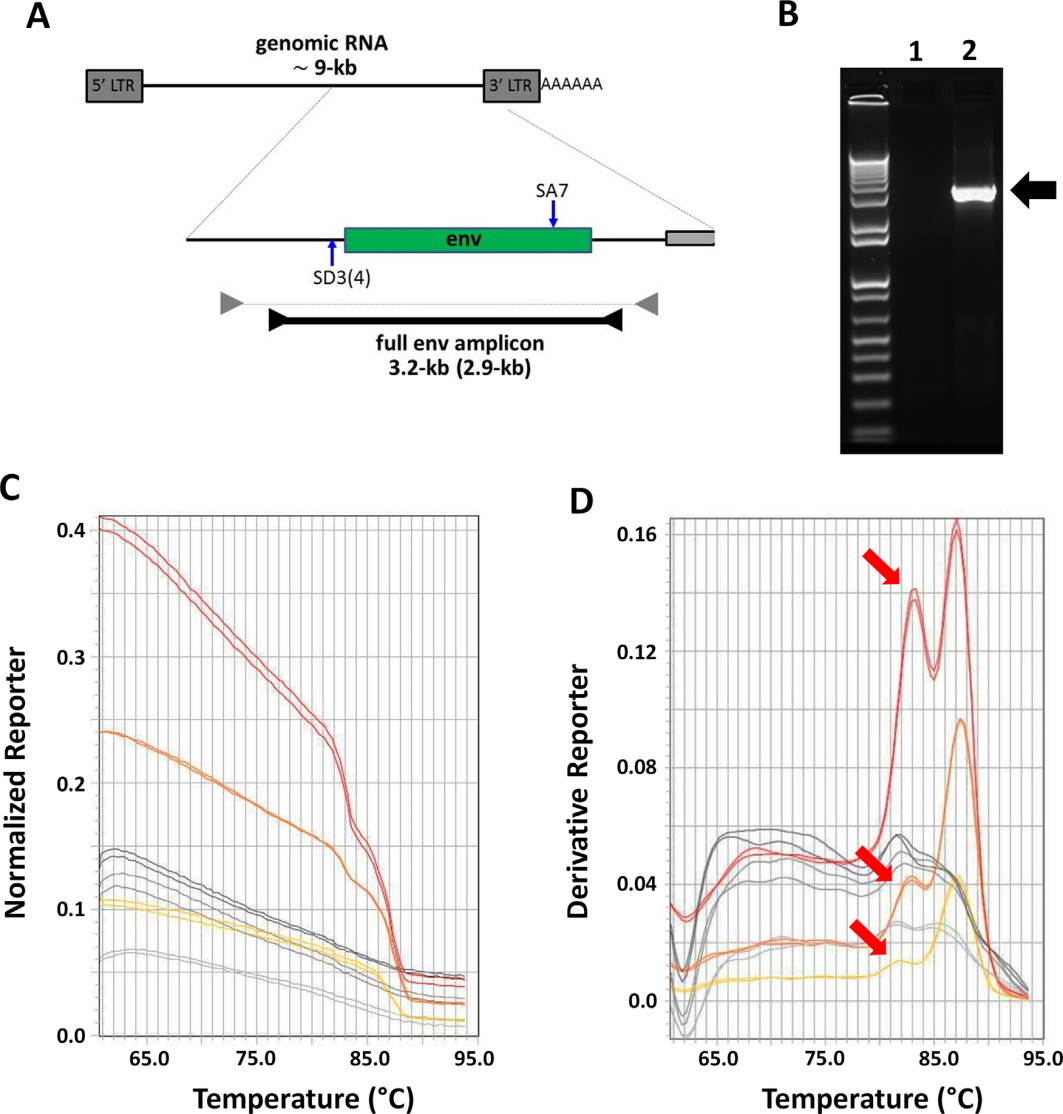
SYBR Green melting profile of the 3.2-kb SIV env SGA-amplicon. (A) Schematic representation of SIV/HIV full env SGA amplicon. The SIV/HIV genomic RNA (top) with a blow-up of env region and the SGA amplicon (bottom) are shown. The relevant splice donor (SD3 for SIV, SD4 for HIV) and splice acceptor (SA7) sites are indicated. The primer pairs used in the first and second run of SGA PCR are represented by grey and black arrowheads respectively. The resulting full env amplicon (3.2-kb for SIV and 2.9-kb for HIV) used for sequencing and phylogenetic analysis is represented. When different from SIV, information relative to HIV is given in parentheses. (B) Gel electrophoresis analysis of the 3.2-kb SIV env SGA-amplicon. 1.2 μl of negative (lane 1) and positive (lane 2) SIV SGA-nested PCR reactions were loaded on a 1.2% agarose gel and stained with fast red to detect the 3.2-kb amplicon (arrow). (C, D) SYBR Green melting analysis of SIV SGA-PCR reactions. 1.2 μl of positive and negative SGA PCR reaction were added to melting reaction mixture containing increasing amounts of SYBR Green and melting run was performed. The resulting melting curves are presented in C and their corresponding melting peaks in D. The shoulder on the lower temperature side is indicated with a red arrow. Yellow, orange and red melting curves/peaks: melting profile of positive SGA reactions with 1, 2 and 4 μl SYBR Green respectively. Light grey, dark grey and Black melting curves/peaks: melting profile of negative reactions with 1, 2 and 4 μl SYBR Green respectively.

A melting analysis of PCR amplification products is usually performed with a low resolution after a standard SYBR Green Quantitative PCR (QPCR) reaction or a high resolution in more specialized High Resolution Melting (HRM) studies, and results in a melting profile specific for the amplicon. Both in the QPCR and HRM context, the melting reaction is performed using the full amplification reaction and amplicons of a maximum length of about 300-bp long. In the widely used SIV env SGA method developed by Keele et al. [1], a very long amplicon (3.2-kb) containing the full length env gene is generated by non quantitative PCR (Fig 1A) and only a small amount of the SGA PCR reaction (typically 1 to 2 μl out of 25 μl) is available for the selection of positive reactions in order to keep the largest amount of material possible for direct sequencing. To assess whether SYBR Green melting reaction could be used to discriminate between positive and negative SIV SGA PCR reactions, using limited amounts of PCR reaction, we added different amounts of SYBR Green mix (1, 2 and 4 μl) to 1.2 μl of a positive and a negative SIV env SGA PCR nested reaction (Fig 1B) in a 8 μl reaction mixture. The reaction was then submitted to a classical melting run. The analysis of the corresponding melting curves revealed that the fluorescence intensity associated with positive PCR samples was always significantly higher than that of negative samples (Fig 1C), with a 2 fold difference for 1 and 2 μl SYBR Green and 3 fold difference for 4 μl SYBR Green (S1A Fig). When examining melting peaks (corresponding to the derivative of the later melting curve), we observed that the 3.2-kb env amplicon present in the positive reaction generated a double peak (Tm 83.2 and 87.0°C) with the amplitude of the peak on the lower temperature side increasing with increased concentrations of SYBR Green (Fig 1D and S1B Fig). Given that gel electrophoresis analysis of the SGA PCR reaction did not show any secondary PCR product (Fig 1B), we speculated that the secondary peak/shoulder most likely resulted from intramolecular properties of the 3.2-kb amplicon. Indeed, several studies have reported that uneven G/C distribution throughout the amplicon can result in shoulders or secondary peaks in the melting profile of a QPCR product [14–16]. A large GC-poor area that could potentially result in a local melting at lower temperature is present in the 3.2-kb amplicon (S2A Fig) and our further results (presented below) support its association with the observed secondary peak/shoulder.

Altogether, these data suggested that initial fluorescence intensity and melting peak profile could be used to discriminate between positive and negative SGA PCR samples using 1.2 μl PCR sample. We chose to work thereafter with 2 μl of SYBR Green corresponding to the lowest amount for a satisfactory difference with background signal both in fluorescence intensity and melting peak profiles (Fig 1C and 1D).

### LAMP screening of SIV full length env-SGA PCR products originated from blood plasma samples

**Fig 2:**
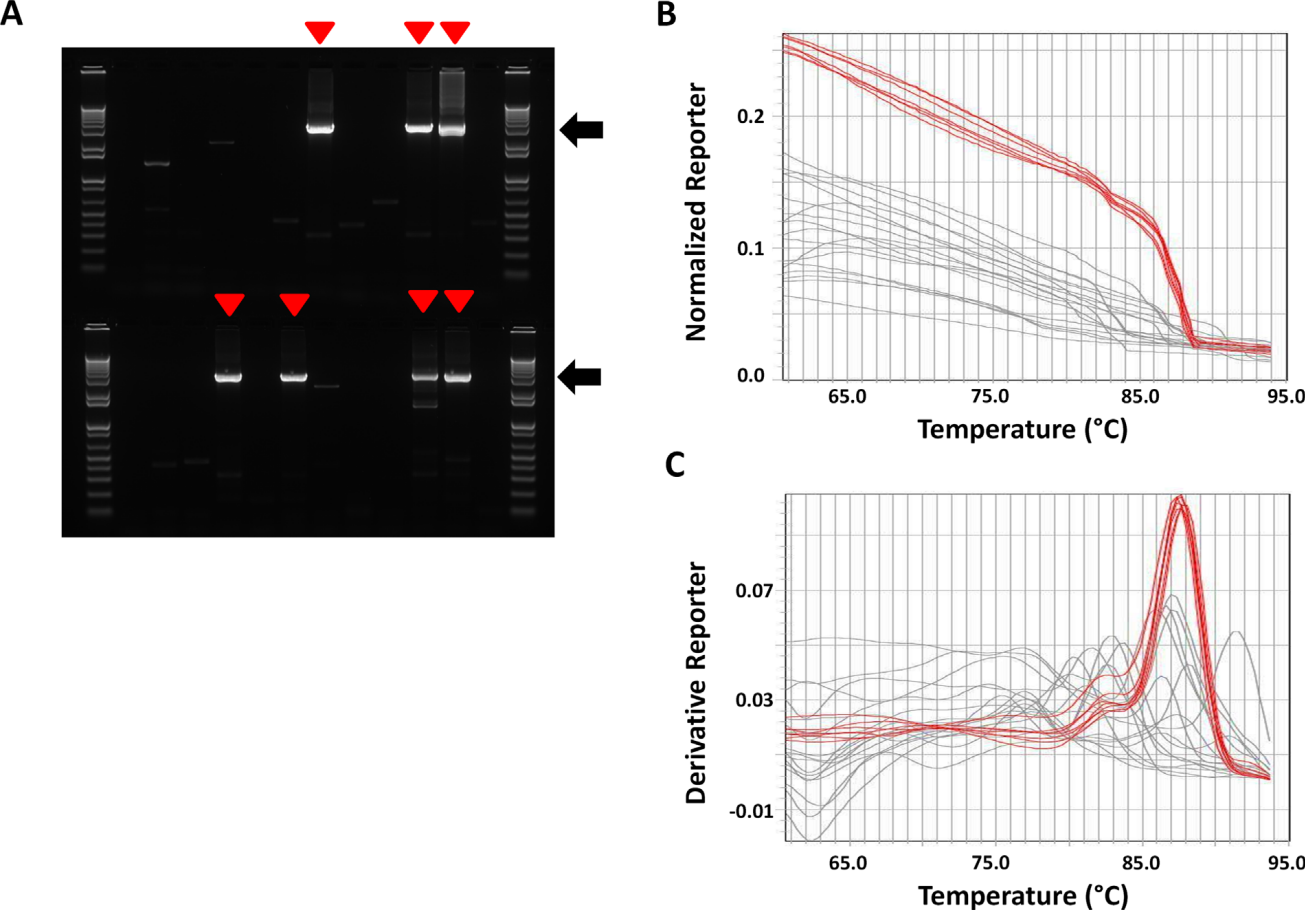
SYBR Green melt profiling of SIV SGA reactions originated from blood plasma sample. RNA was extracted from the blood plasma of an SIV infected macaque and used for cDNA synthesis. 24 SIV SGA PCR reactions were performed starting from the RT reaction using a working dilution of 1/33. (A) Agarose gel analysis of 24 SGA reactions. 1.2 μl of the 24 SGA reactions were loaded on 1.2% agarose gel. The positive reactions containing the 3.2-kb env amplicon (black arrow) are indicated with a red arrowhead. (B, C) SYBR Green melting analysis of the 24 SGA reactions. Melting reactions were performed using 1.2 μl of the SGA reactions shown in A and 2 μl of SYBR Green in a total volume of 8 μl. The graphs corresponding to the melting curves and the melting peaks are given in B and C respectively. The red curves are corresponding to the 5 positive reactions indicated in A.

To further validate the use of LAMP for the detection of SIV-SGA positive reactions, SIV SGA PCR reactions were carried out using cDNA corresponding to blood plasma RNA samples collected from an SIV infected macaque and analyzed using both agarose gel electrophoresis and SYBR Green melting. The same amount of PCR sample (1.2 μl) was used for both analyses. The melting reaction was performed with 2 μl of SYBR Green. Gel analysis revealed 7 out of 24 SGA PCR reactions positive with a unique 3.2-kb band corresponding to the full env amplicon, and 17 negative reactions displaying smaller band sizes with low intensity (Fig 2A). Accordingly, the melting analysis showed higher fluorescence intensity levels and melting peaks with a shoulder on the lower temperature side for all 7 positive samples identified by gel analysis (Fig 2B and 2C). Thus, these results demonstrate that LAMP accurately identifies full length SIV env amplified from blood plasma by SGA.

### LAMP screening of SIV full length env-SGA PCR products originated from infected cells, organs, and additional body fluid

**Fig 3:**
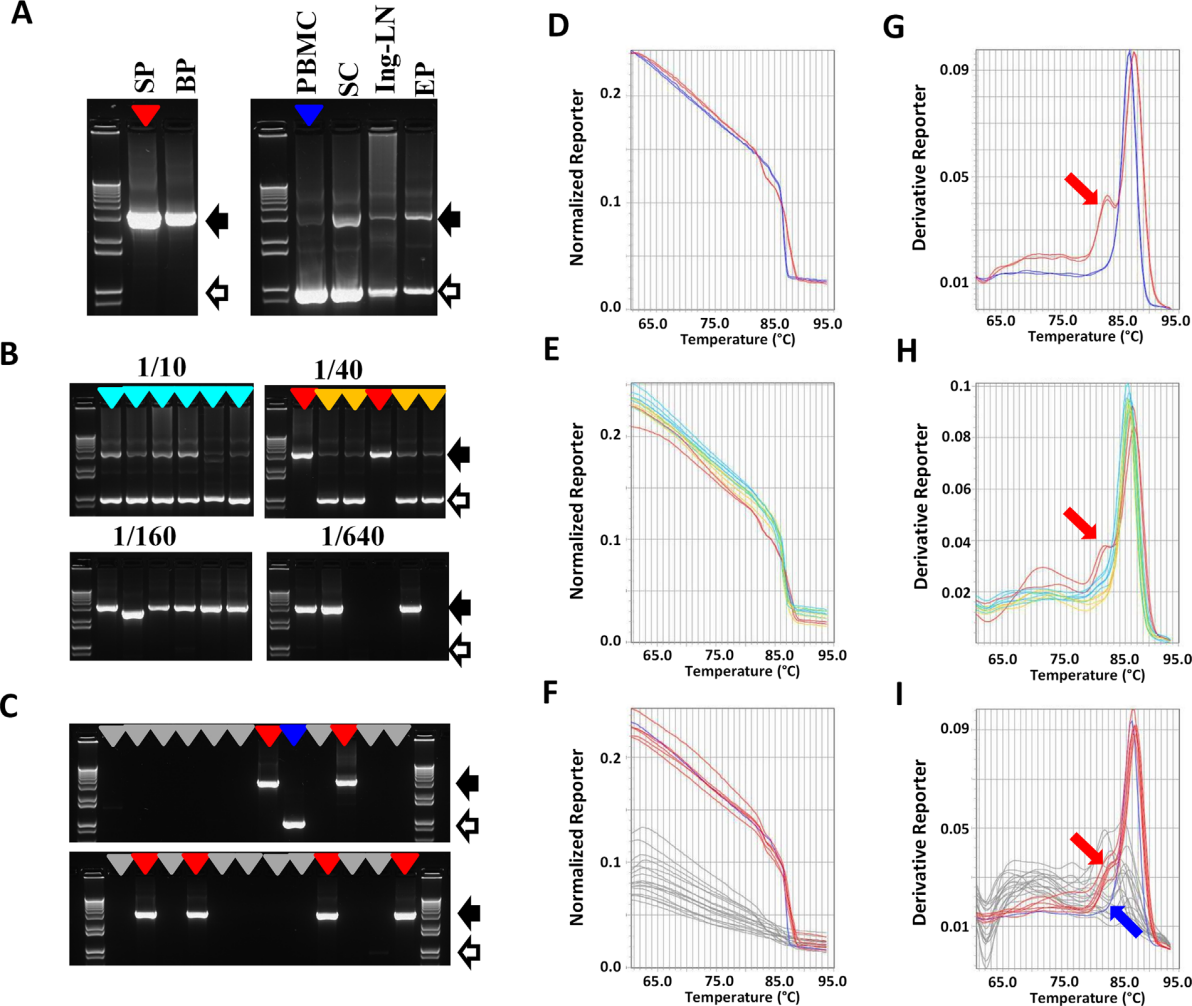
SYBR Green melting profile of SGA reactions originated from cell and tissue samples. (A,B,C) Gel electrophoresis analysis of SGA reactions originated from cell and tissue samples. 1.2 μl of SIV env SGA PCR products obtained using undiluted cDNAs from body fluids, cells and tissues (A), serial dilutions of inguinal node cDNA (4x serial dilution from 1/10 to 1/640, 6 replicates each dilution) (B) and cDNAs from semen cells at SGA working dilution (24 replicates) (C), all originated from an SIV infected macaque, was analyzed on 1.2% agarose gel. The 3.2-kb full env and 950-bp MS RNA amplicons are indicated with black and open arrows respectively. Arrowheads in A, B and C indicate the samples used for the melting profiles shown in D–I. D–I) SYBR Green melting profile of SGA reactions originated from cell and tissue samples. The melting curves (D–F) and melting peaks (G–I) corresponding to the samples indicated with the colored arrowheads in A, B and C are given in D and G, E and H, F and I respectively. Melting reactions were performed using 1.2 μl of SGA reaction mixture. The same color is used for arrowhead, melting curve and melting peak of a given sample (red: 3.2-kb env amplicon; dark blue: 950-bp MS RNA amplicon; light blue and orange: mix of amplicons from reactions performed at 1/10 and 1/40 cDNA dilutions respectively; grey: negative reaction). The shoulder on lower Temperature side specific for the full env amplicon is indicated with a red arrow. SP: seminal plasma; BP: blood plasma; PBMC: peripheral blood monocytes; SC: semen cells; Ing-LN: Inguinal Lymph node; EP: epididymis.

To take into account that SGA can be used to study viral compartmentalization in different organs, cell types and body fluids, we decided to further validate the use of the SYBR Green melt profiling using samples from various sources. Unlike with body fluid samples, the gel electrophoresis profile of SGA PCR reactions performed using undiluted RT reactions corresponding to infected organs and cells showed a dominant 950-bp product corresponding to a multi-spliced (MS) RNA derived amplicon, as determined through sequencing, and only small amount, or lack of full length SIV env amplicon (Fig 3A and S2A Fig). When approaching the limiting dilution condition, the frequency of detection of the 950-bp product decreased, whereas the frequency of detection of the 3.2-kb product increased (Fig 3B and 3C), probably due to lower amount of SIV MS RNA compared to the SIV full length genomic RNA in the initial sample. However, although, the 3.2-kb product was more frequent than the 950-bp product when working at SGA limiting dilution, MS RNA amplicon could still be detected in a small subset of SGA PCR reactions (about 30% in some tissues). Therefore it was necessary to be able to detect reactions encompassing MS RNA products in order to exclude them from the sequencing step. In order to assess whether LAMP may be used in this context, we compared the melting profile of both amplicons. We carried out melting reactions with 1.2 μl of either 3.2-kb or 950-bp amplicon added to SYBR Green melting mix. The resulting melting curves showed no consistent differences between intensity levels (Fig 3D). However, melting peaks analysis revealed that, unlike the 3.2-kb amplicon, no shoulder at low Tm was observed with the 950-bp amplicon (Fig 3G). This result further indicated (see section on SYBR Green melting analysis of the 3.2-kb SIV env SGA-amplicon) that the existence of a shoulder at a lower Tm constituted a signature specific for the 3.2-kb full env amplicon, likely due to the local melting of a GC poor intramolecular region. Since this region is located in the SD3-SA7 intron, it is lacking in the MS RNA derived amplicons (S2A Fig), which indeed showed no shoulder. Thus these data demonstrated that LAMP allows the discrimination between spliced and unspliced env RNA amplicons.

To validate further the use of LAMP for SGA studies using organs and cells, we performed the melt profiling of PCR reactions corresponding to the cDNA serial dilution and SGA testing used in the gel analysis previously presented in Fig 3B and 3C respectively. Melting curve analysis showed that, as expected, no difference was observed in intensity levels between PCR reactions corresponding to the 1/10 and 1/40 RT dilution samples (Fig 3E). On the other hand, melting peak analysis showed that the profiles corresponding to the 2 reactions containing the 3.2-kb PCR product were easily detected due to the presence of the shoulder (Fig 3H). These results indicate that LAMP can also be used on RT serial dilutions to determine SGA working dilution. The melting analysis of the 24 SGA reactions showed that, as expected, a difference in intensity levels was observed between amplicon positive and amplicon negative reactions (Fig 3F), and that discrimination between full env and MS RNA derived amplicons was possible using the shoulder specific for the 3.2-kb PCR product (Fig 3I). Taken together, these results demonstrate that LAMP can be used for profiling SGA PCR reactions using infected cells and organs.

### LAMP screening of HIV full length env-SGA PCR reactions

**Fig 4:**
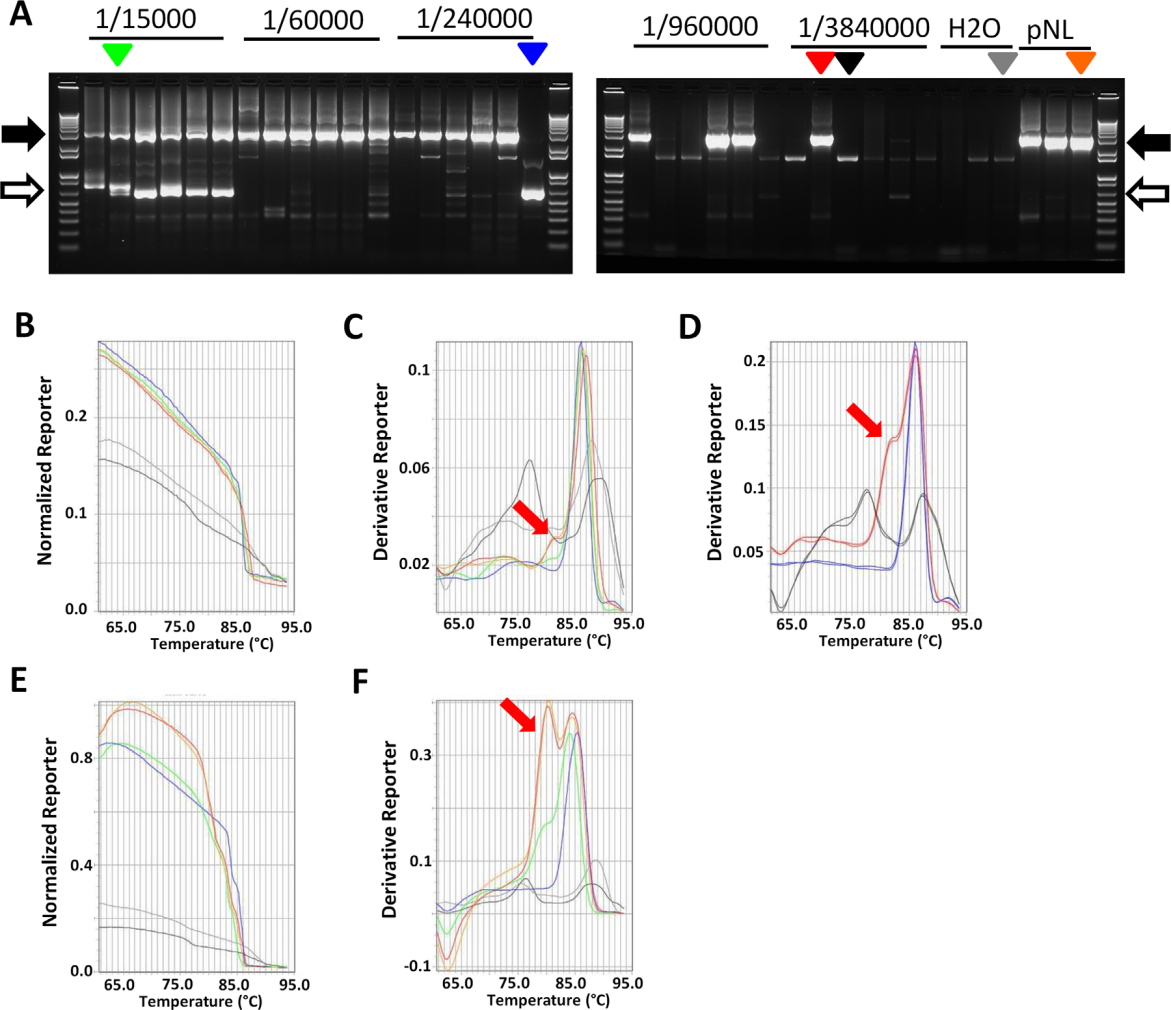
Melting analysis of HIV SGA-amplicons. RNA extracted from Jurkat cells infected with HIV-1 (IIIB strain) for 8 days was used for cDNA synthesis. HIV cDNAs were quantified using QPCR to determine the SGA working dilution. Next, HIV SGA-PCR was performed using RT serial dilution in a range including the SGA working dilution (6 PCR reactions for each dilution). SGA-PCR performed using 20 copies of pNL-4.3 HIV molecular clone was used as a positive control for full env amplicon. (A) Agarose gel analysis of HIV SGA-PCR products. 1.2 μl of each PCR reaction was loaded on a 1.2% agarose gel and stained with GelRed dye. The 2.9-kb full env and 650-bp MS RNA amplicons are indicated with black and open arrows respectively. Arrowheads are used to show examples of amplification profiles (red and orange for the 2.9-kb full env product amplified from Jurkat cDNA and pNL4.3 plasmid respectively; blue for the 650-bp amplicon corresponding to the MS HIV RNA amplification product; green for a mix of full env and spliced RNA amplification products; black and grey for profiles corresponding to negative reactions). (B,C,D) SYBR Green melting analysis of HIV SGA PCR products. SYBR Green melting reactions were performed using 2 μl (B,C) or 4 μl (D) SYBR Green mix reagent and 1.2 μl of the PCR reactions indicated with arrowheads in A and the resulting melting curves and/or melting peaks are shown. The colors used for melting curves correspond to the one used for arrowheads in A. The shoulder on the low temperature side specific for full env amplification product is indicated with a red arrow. (E,F) EvaGreen melting analysis of HIV SGA PCR products. Melting reactions were performed as indicated for B and C using EvaGreen dye instead of SYBR Green.

The results presented above showed that LAMP is a good alternative to gel electrophoresis for SGA studies performed using SIV infected cells, organs and fluids. We then tested whether this method could be extended to HIV sequence analysis based on full env SGA. We used the HIV env SGA protocol developed by Keele et al. [3] and RNA samples extracted from HIV infected Jurkat cells. We first performed SGA PCR reactions starting from serial dilutions of RT reactions and analyzed the amplification profiles by loading on agarose gel. As with SIV, we observed that, at a low dilution factor, the 2.9-kb/full env amplicon was associated with smaller amplicons (from 650 to 800-bp), whereas a unique amplification product was observed at higher dilutions (Fig 4A). Considering the sizes of these smaller amplicons, the primers used and splicing site positions, HIV MS RNAs were most likely the source of these amplicons, similarly to what we observed with SIV (Fig 1A). We next tested the use of the SYBR Green melting method as a tool to analyze HIV SGA PCR products. Samples corresponding to PCR reactions with different gel profiles and/or matrixes were selected and submitted to LAMP using the same experimental conditions as for SIV melting. Melting curve analysis showed that, as expected, all the amplicon positive samples corresponding to either full env, MS HIV or mixed amplicons, showed a similar fluorescence intensity level, which was higher than that observed for negative samples (Fig 4B). Melting peaks comparison showed that, as with SIV, a shoulder on the low temperature side was observed specifically for the full HIV env amplicon (Fig 4C). Similarly to SIV env, HIV env encompasses a GC poor area, most likely responsible for the amplicon specific signature observed (S2B Fig). Taken together, these results demonstrated that HIV MS RNA can compete with genomic RNA for SGA amplification, but, as we previously observed with SIV samples, working at limiting dilution will be in favor of the 2.9-kb amplicon. Moreover, we also showed that LAMP allowed discrimination between SGA positive and negative reactions using fluorescence intensity and between full env and MS RNA amplicons using the melting peak profile. However, when compared with SIV melt profiling, we observed that these differences were less marked with HIV SGA (compare Fig 4B and 4C to Fig 1C and 1D). Increasing SYBR Green amounts further supported this observation (S3 Fig) and showed that to facilitate the detection of the specific unspliced HIV env product over spliced env products, a higher amount of SYBR Green (4 μl) was necessary (Fig 4D). In order to find a way to keep the method at low cost, we tested the use of the EvaGreen Dye, which has a cost similar to SYBR Green and a higher sensitivity. The reason why this dye was not initially chosen in our tests is that it is not as yet as widely used as SYBR Green in standard laboratories. The melting profile obtained using 2 μl of EvaGreen compared with that obtained with 2 μl or even 4 μl of SYBR Green showed a significant increase in both the differences in intensity between positive and negative samples and the differences in peak profile between full env and MS RNA amplicons (compare Fig 4E with 4B and Fig 4F with 4C and 4D; S3 Fig). Therefore, EvaGreen appeared as a more powerful and sensitive SGA profiling tool than SYBR Green and is more convenient when working with HIV env SGA amplicons.

### Examples of strategies for large scale LAMP analysis for HIV and SIV full length env SGA

**Fig 5:**
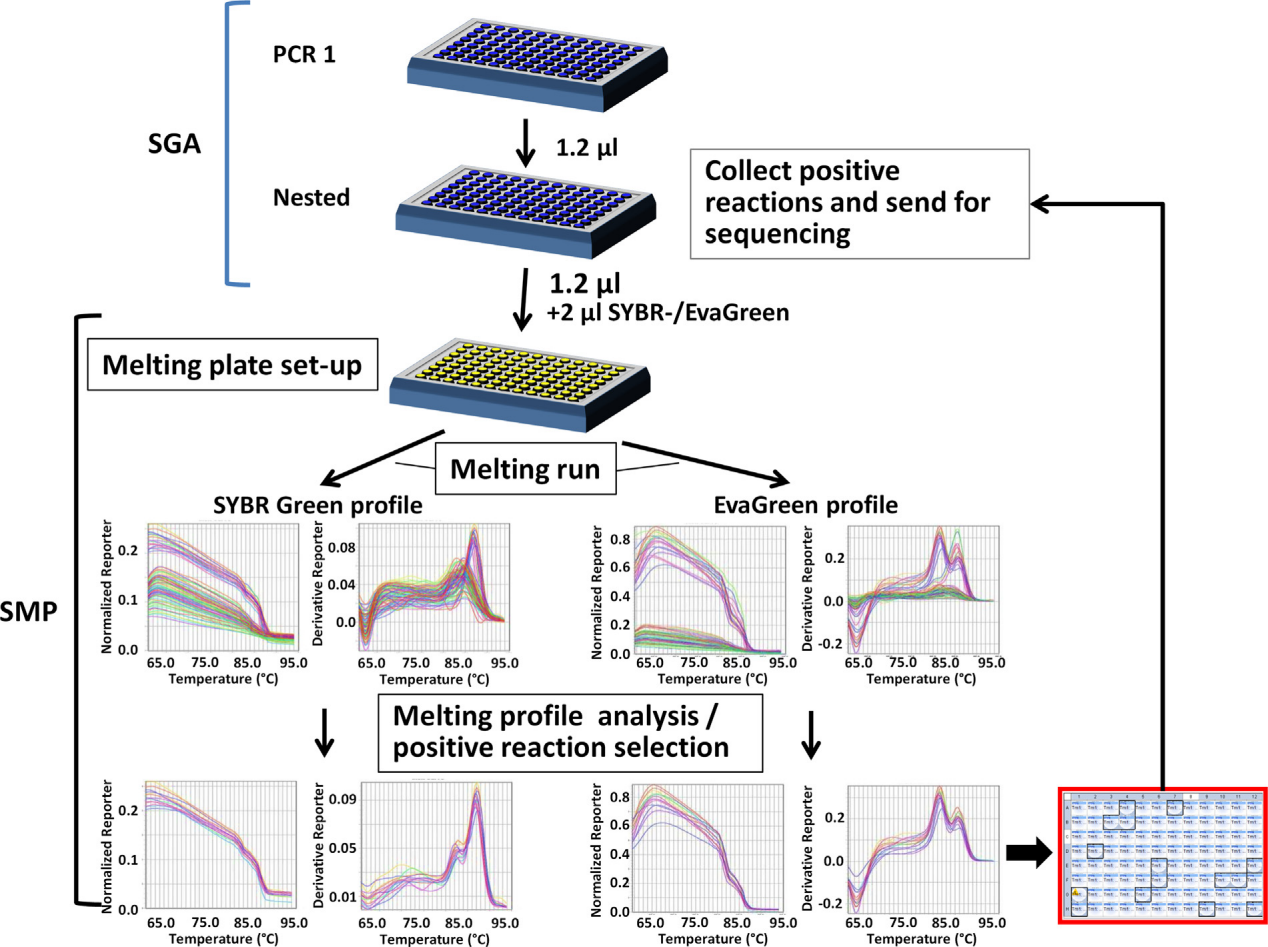
96 well scale screening of SIV env SGA-amplicons using SYBR Green or EvaGreen melt profiling. cDNA synthesized from RNA extracted from semen cells of SIV infected macaque was used to perform 96 SIV SGA PCR reactions in a 96-well plate using a SGA working dilution of 1/34. 1.2 μl of SGA nested reactions was transferred in 96well plates containing SYBR Green or EvaGreen melting reaction mixture and melting run was performed. The resulting melting curve and melting peak profiles of the full plate (top graphs) were analyzed to isolate the 3.2-kb full env amplicon specific melting profile (bottom graphs) and map the corresponding wells on the plate (red outlined plate). The corresponding SGA positive reactions were collected on the nested PCR plate and sent for sequencing.

**Fig 6:**
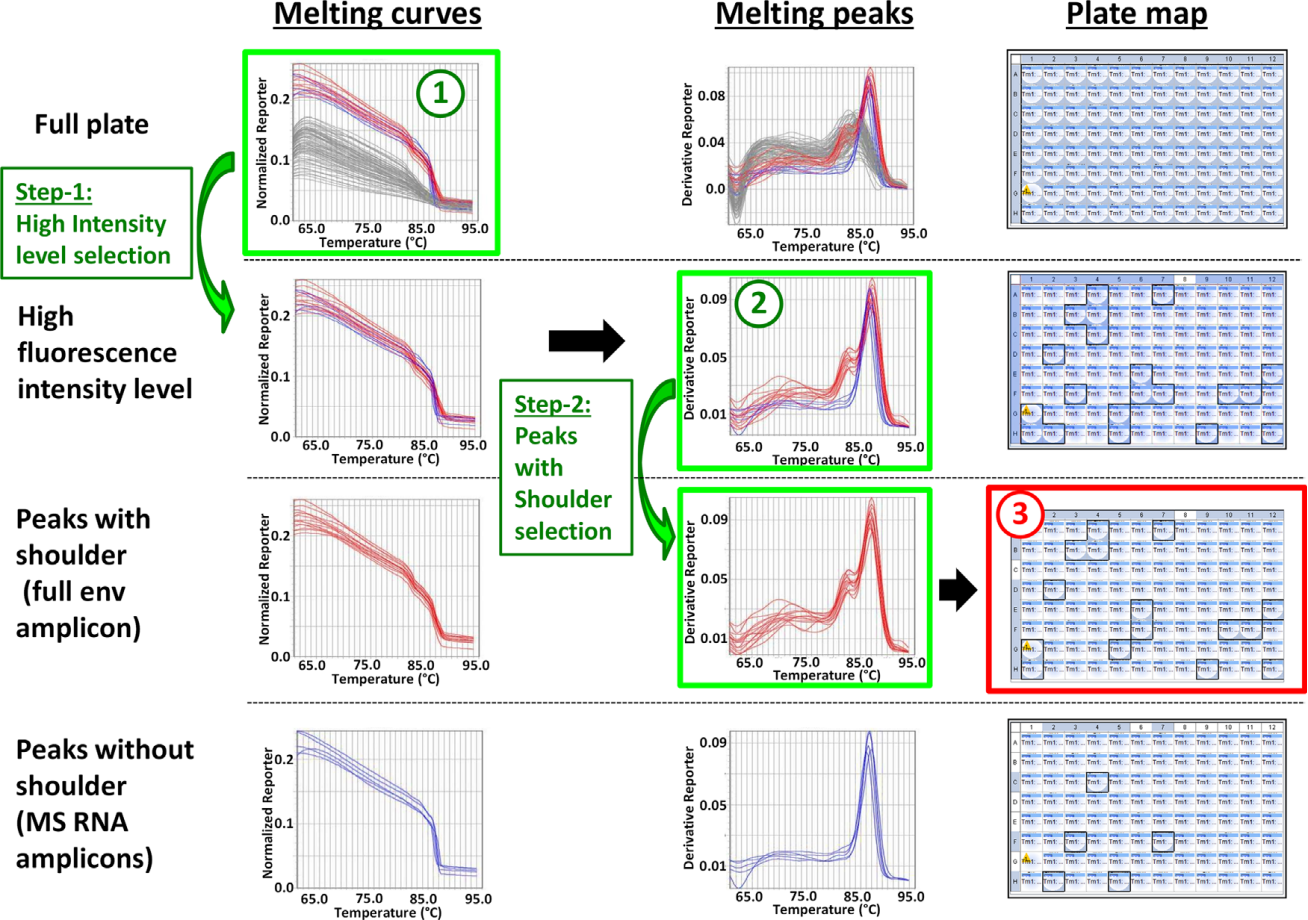
Analysis of a 96-well plate SIV SGA SYBR Green melting profile using the “Graph-2steps” method. From the full plate SIV SGA SYBR Green melting curve profile (1), the curves with higher intensity level corresponding to the presence of an amplification product are selected (step 1). From the corresponding melting peak profile (2), the peaks with a shoulder (red curves) specific for the 3.2-kb full env amplicon are selected (step 2) and the corresponding wells located on the plate (3). The peaks in blue excluded in step 2 correspond to the MS RNA amplicon. This 2 steps graphical method can be used with any fluorescent dye and for both HIV and SIV SGA with most softwares and will require between 3 and 4 minutes for a 96well plate.

Since the most widely used format for PCR is 96-well plates, we started by performing 96 SGA PCR reactions using cDNA from SIV infected semen. 1.2 μl of the nested reactions were then used to perform SYBR Green and EvaGreen melt profiling (Fig 5). The resulting melting profiles showed that, as mentioned above, EvaGreen profiles had a more pronounced separation between groups of different fluorescence intensities and of different peak profiles (Fig 5, top graphs). However in both cases, the 3.2-kb full env amplicon specific melting profile was easy to isolate from negative reactions or other products (Fig 5, bottom graphs). Three different methods for large scale LAMP analysis were developed. The first method is called the “Graph-2steps” method and can be used for SIV-SYBR and SIV/HIV-EvaGreen SGA profiling with any QPCR analysis software. In the first step, the curves with higher intensity levels are selected starting from the lane A to end up with the full plate. In the second step, the melting peaks corresponding to the selected wells are visualized one by one by using unclick/click process to select the one with a shoulder corresponding to the 3.2-kb full env amplicon and get their corresponding location on the plate map (Fig 6). We successfully used the “Graph-2steps” method in the context of a large scale SIV SGA study, both to determine the appropriate dilution of cDNA for SGA and to screen for SGA positive reactions. Using this method, we screened over 5000 SIV SGA PCR reactions, corresponding to samples originating from 2 different body fluids (blood and semen), 2 cell types and 5 different organs isolated from SIV infected macaques (Table 1) and isolated 685 full env amplicons using SYBR Green melt profiling, which were double checked using gel electrophoresis. Gel analysis revealed that 679 amplicons (99%) were indeed full length env amplicons, while 6 amplicons represented fragments between 2 and 3-kb most likely corresponding to deletion containing fragments. 557 amplicons were sequenced, which showed that 486 (87.3%) originated from a single cDNA molecule as demonstrated by the absence of double peaks on chromatograms. This ratio is in accordance with the expected Poisson distribution of about 80% [4].

**Table 1:** Validation of SYBR Green LAMP in a large scale SIV SGA study.

SIV SGA PCR screened using LAMP	Env amplicon positive reactions	Gel analysis validation	Sequence analysis validation
5068	685	679/685 (99%)	486/557 (87.3%)

To validate the Graph-2steps method for HIV-SGA studies, we performed 96 HIV SGA PCR reactions with a sample corresponding to cDNA from HIV infected Jurkat cells followed by an EvaGreen melt profiling. Of note is that Eva green HIV melting curves and melting peak profiles presented a greater complexity than the one obtained for SIV using the same dye (compare S4 Fig with Fig 6). Gel analysis and melting profiles comparison showed that this increased complexity resulted from the presence of six amplicons with a size range between 1 and 2.5-kb in addition to the five 650-bp MS RNA amplicons (S5 Fig, left panels). However, this did not impair the selection of the full length amplicons and thanks to their specific melting peak profile, 23 samples positive for env full amplicon were readily selected out of 62 negative reactions and 11 reactions with unspecific products (S4 Fig). Depending on the number of positive reactions, the time for a full plate analysis using the Graph-2steps method will last between 10 seconds for one positive sample to 3 to 4 minutes for a plate containing around 30 positive samples.

An alternative to the above graphical method is the “Excel sheet/values” method using the fluorescence intensity and Tm values corresponding to the melting profile (S6 Fig). This method requires first to extract the raw data (intensity values for each reading point) and results (peak Tm values) in order to elaborate an Excel working sheet. This working sheet will contain for each of the 96 wells the fluorescent dye and passive reference (if any) intensity values from a reading point close to the beginning (here reading 10) and the corresponding values of the melting peaks Tm1 and Tm2 identified by the software. The ratio fluorescent dye/passive reference, corresponding to the best approximation of the corrected values plotted in the intensity graph, will be calculated in a separate column. Then applying filters for higher ratio intensity and lower Tm will result in the selection of positive samples. This method can be used for both SIV and HIV SGA analysis but with EvaGreen melting profile only since a high resolution in intensity and Tm is needed. The method will take a bit longer than the previous one, around 5 to 8 minutes, most of the time corresponding to the extraction and working sheet elaboration, the filtering lasting only few seconds. However, the strong advantage of this method is that the time spent on the QPCR instrument software is very short and that, because of the very short time needed for the data filtering step, its lasting time will be very minimally affected by an increase in the data scale, making it very interesting for the analysis of several 96 well plates simultaneously. Finally, the last method, the “Graph-1click” method, is by far the most efficient one, with a time laps of few seconds for 96-well scale analysis. This method can be used for SIV-SYBR and SIV/HIV-EvaGreen SGA profiling and consists in directly clicking on the melting curves and/or melting peaks to select and/or exclude the one corresponding to positive and negative samples respectively. A restriction however is that not all onboard QPCR instrument softwares enable the selection of wells through direct clicking on the corresponding curves. The example of SIV-EvaGreen profile analysis shown in S7 Fig is a one click process and lasts less than 5 seconds.

**Fig 7:**
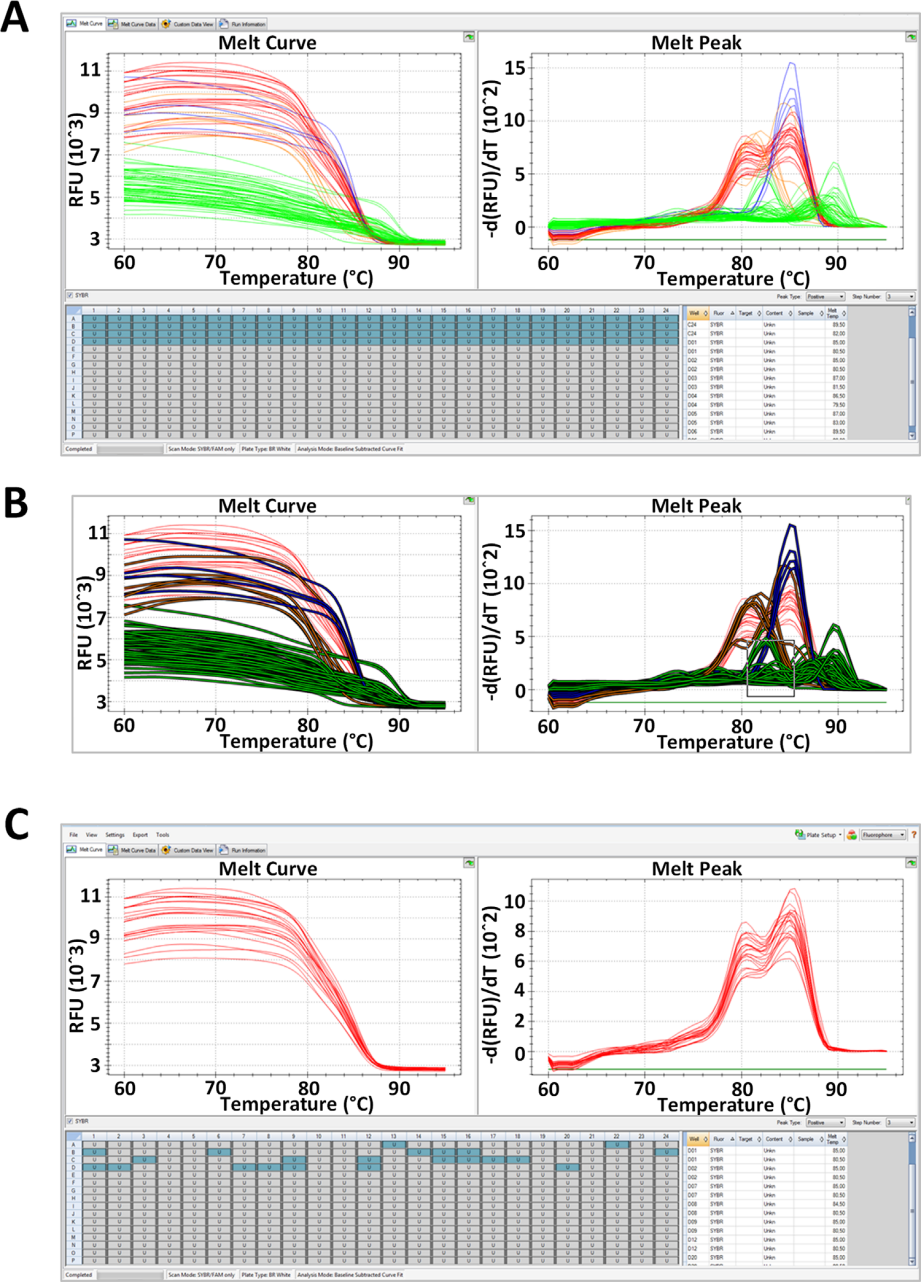
LAMP screening of HIV full length env-SGA PCR reactions using a 384-well plate. 1.2 μl of 96 HIV env SGA nested reactions were transferred in a 384-well plate containing the melting mix (EvaGreen dye) and melting run was performed on a CFX384 Touch Real time PCR detection system instrument (Bio-Rad). The melting curve and melting peak profiles were similar to the one observed under 96-well format showing differences in fluorescence intensity and/or peak shape between negative reactions (light green curves) and env (red curves), spliced RNA (blue curves) or other amplicon (orange curves) containing reactions (A). Melting profile was analyzed using the fast “Graph-1click” method by selecting the melting peaks corresponding to the negative and unwanted product containing reactions (B) in order to exclude them and thus map the remaining positive reaction profiles on the plate (C).

We assessed whether the methods we developed using the 7500 real Time PCR System instrument (Applied Biosystems) for 96 well plates could be up scaled using a 384-well Real time PCR instrument to allow the profiling of SGA PCR performed in 384-well format or up to four 96-well SGA plates at once. We used a CFX384 Touch real time PCR system instrument (Bio-Rad) to carry out SYBR Green and EvaGreen melt profiling of the same 96-well SIV SGA reactions analyzed used previously. The results showed that discrimination of SIV env 3.2-kb amplicon positive reactions using amplification curve and melting peak profiles was achievable with both dyes using this format (S8 Fig). Similarly, we successfully isolated the 2.9-kb HIV amplicon using the melting profile of HIV SGA reactions performed with the 384-well instrument (Fig 7). The onboard software present on the CFX384 Touch real time PCR system instrument allowed locating the positive reactions in a few seconds using the “Graph-1click” method. The time for analysis will be similar for a full 384 plate analysis, making the “Graph-1click” method extremely efficient for 384-well plate analysis. However, as mentioned previously, this method is not compatible with every onboard software. While the “Graph-2steps” method is compatible with any software and any dye, the time for analysis will increase from 3–4 min for a 96w to 12–16 min for a 384w. As a result, the “Excel sheet/values” method will be interesting when using the 384-well format since, as mentioned previously, its time for analysis will remain the same, between 5 and 8 minutes for a full 384-plate analysis. The characteristics of the 3 methods described above are summarized in Table 2.

**Table 2:** Characteristics of the different methods used for melting profile analysis.

Method	Compatibility			Maximum analysis time (min)*		Plus/minus points
	SIV		HIV			
	SYB	Eva	Eva	96-well	384-well	
**Graph-2 steps**	+	+	+	3–4	12–16	(+) large compatibility
						(-) long analysis in 384w format
**Graph-1 click** **	+	+	+	<1	<1	(+) fast analysis
						(-) software restriction
**Excel sheet / values**		+	+	5–8	5–8	(+) fast analysis for large scale data; limited time spent on onboard software
						(-) The longest for 96w scale; Eva restricted

* The duration of the analysis will depend on the number of positive samples to select and will range from 0 min for a plate with no positive samples to the maximum analysis time indicated there for a plate with 30% of positive reactions

** The graphical one click analysis method requires a software with a graphical curve selection capacity (Biorad CFX manager for example)

SYB: SYBR Green; Eva: EvaGreen

In conclusion, a typical LAMP experiment will last less than one hour in total for the screening of one 96- or 384-well plate, including:—a 10 to 20 minutes long experiment set up corresponding to a simple plate to plate sample transfer;—a melting run of 40 minutes maximum (depending on the instrument);—an analytical time comprised between 10 seconds and 8 minutes for a 96 well format, and 10 seconds and 16 minutes for a 384 well format experiment, depending on the method used for analysis (S1 Table). In comparison, gel electrophoresis assay will last 2 to 5 hours approximately to screen one 96- or 384-plate respectively using a standard 40-well gel system, reflecting the time involved in preparing, loading and running the numerous gels required. Altogether, the screening of the 5000 SGA nested PCR that we mentioned earlier would require to perform over 130 gels for electrophoresis analysis and therefore would constitute a highly labor intensive and tedious step in the study which will generate lots of chemical waste. In conclusion, LAMP will make large scale SGA studies more accessible to standard laboratories by saving time and effort at affordable costs.

## Discussion

Studies using SGA to assess virus genetic diversity typically require the screening of several thousands of PCR reactions to isolate positive samples that should not exceed a representation of 30% to guaranty single molecule amplification. This step is traditionally performed using a classical agarose electrophoresis analysis and constitutes a very labor-intensive step in most laboratories which do not have access to high throughput DNA electrophoresis system. The method described here enabled to replace gel electrophoresis analysis with SYBR Green or EVA Green melting curve analysis of HIV/SIV env amplicons, which is faster (total of about one hour for screening 384 samples) and easier to handle in large scale studies due to minimal processing steps. This method is accessible to all laboratories performing QPCR as well as cost effective due to the use of only limited amounts fluorescent reagent.

Genetic differences between virus populations present in different tissues in a given individual, referred as virus compartmentalization, have been reported in many studies (reviewed in [17, 18]). As a result, characterization of blood plasma virus population will only constitute a partial knowledge of the full genetic panel present in an infected patient. Consequently, many studies are now focusing on virus populations present in other body fluids (semen, urine, saliva, cerebrospinal fluid, milk) and/or organs (brain, genital tract, gut, mammary glands…). Analysis of virus genetic diversity in infected cells/organs can be performed using either RNA or DNA extracted samples. Unlike the genetic profile obtained from viral RNA, the one corresponding to viral DNA will also include sequences corresponding to archived unexpressed proviral DNA present in latent cells or defective viral DNA. Therefore RNA and DNA derived profiles can be different and the choice between them will depend on the aim of the study. For example, genetic profile via DNA will be necessary to obtain a more complete panel of all the quasispecies present, independently of their expression, whereas a profile derived from RNA will be better suited to track the tissue or cellular source of a population of free virus particles. LAMP can be used to screen SGA reactions performed from DNA and RNA samples. However, both for SIV and HIV SGA, performing SGA using organ/cell RNA samples can result in smaller size PCR products corresponding to MS RNA and /or deleted amplicons that can reach 30 to 50% of all amplicons and should be removed from the study before the sequencing step. We showed here that the full env amplicon can be discriminated from the MS RNA amplicon using LAMP due to differences in their corresponding melting peaks.

We present three different methods, namely the “Graph-2steps”, the “Excel sheet/values” and the “Graph-1click” methods, to analyze the melting profiles and locate the wells positive for full env amplicon. The 2 steps method will be compatible with both SIV and HIV studies and with any dye on any QPCR instrument. However, given that it takes about 4 minutes to analyze the melting profile of a full 96w plate, and about 16 minutes for a 384w plate, this method will not be the most appropriate for large scale screenings. With an analyzing time of less than one minute for both 96 and 384 well formats, the 1 click method will be preferred for large scale SIV and HIV studies. However, this method will require the ability to select the melting curves by clicking on the graphical interface, a function that is present on some of the common QPCR analysis softwares but not all. Last, we present a third method no more based on graphical interface but on raw fluorescent intensity and Tm value filtering using excel software. This method can be very useful when access to QPCR software is restricted. Given that the most time consuming step is extraction of the raw data and elaboration of excel worksheet, this method will be more time-efficient for large scale studies. It is compatible with both HIV and SIV studies and with any QPCR software with an access to raw data. However, the use of EvaGreen will be necessary to have a higher potential for discrimination between melting curves and melting peaks.

Our report was focused on full env amplicon since this is the longest, the most informative and most used amplicon for phylogenetic analysis. However, other areas in SIV and HIV genomes have been used, like gag or pol genes. It is most likely that the LAMP method herein described will be compatible with such studies since our work showed that any PCR positive reaction, independently of the size of the amplicon, can be discriminated against negative reaction using melting curve and/or peak profile. Moreover, considering the splicing pattern of HIV and SIV viruses, working in other areas than env gene should prevent interference of spliced RNA in target sequence amplification. In conclusion, the LAMP approach and the different analyzing methods described here represent a rapid and cost effective alternative to the labor-intensive and time consuming gel electrophoresis based method for large scale SIV/HIV SGA studies involving large number of patients, tissues or samples with very low infection level. In fact, melt profiling could replace the tedious gel electrophoresis analysis in any large scale screening of positive PCR reactions, such as screening for rare mouse genotype or plasmid variants in cloning studies.

## Supporting Information

S1 Fig Effect of increasing amounts of SYBR Green on fluorescence intensity and melting peak profile of positive and negative SGA reactions. (A) Effect of increasing amount of SYBR Green on relative fluorescence intensity between positive and negative reactions. The raw data were extracted from the melting run presented in Fig 1 and used to calculate the corrected initial fluorescence intensity values (SYBR Green signal intensity/ROX signal intensity for each sample, using the intensity values from the first reading) corresponding to the best approximation of the plotted values. The ratio between the values corresponding to positive and negative reactions was calculated for each dose of SYBR Green and expressed as a fold increase in fluorescence intensity. (B) Effect of increasing amount of SYBR Green on melting peak profile of the 3.2-kb SIV env SGA-amplicon. The melting peaks obtained for 1.2 μl SGA PCR reaction melted with 1 μl (left panel), 2 μl (middle panel) and 4 μl (right panel) SYBR Green are represented separately to highlight the relative height between the major and minor (red arrow) peaks. The peaks detected by the onboard software are indicated with a blue dashed line and the corresponding Tm values are given in blue.
(TIF)

S2 Fig Schematic representation of SIV and HIV SGA amplicons. (A) SIV SGA full env and MS RNA amplicons. The %GC (heat map), Env protein ORF (dark arrow), V1 to V5 variable regions (grey boxes) and gp120/gp41 junction (double headed arrow) present in the 3.2-kb full env amplicon (red line, and red arrows for primers) are shown. The blue boxes, corresponding to the region of the 950-bp amplicon sequenced using the primer indicated as a blue arrowhead, are represented above their corresponding part in the full env amplicon and correspond to a junction between the donor splicing site SD3 and the acceptor splicing site SA7. A similar junction was observed in the sequence called “SIV tat and rev genes, complete cds” corresponding to one of the SIV MS RNA and represented as a grey box. The oval delimited with a red line corresponds to the GC poor area present in the SD3-SA7 intron and most likely responsible for the shoulder on low temperature side specific for the full env amplicon. (B) HIV SGA full env amplicon. Similar to SIV, HIV amplicon show a GC poor region (red oval) located in the SD4-SA7 intron.
(TIF)

S3 Fig SYBR and Eva-Green Melting profile of SIV and HIV env and MS RNA SGA-amplicons. Melting reactions were performed using 1.2 μl of a SGA PCR reaction containing the SIV or HIV env or MS RNA SGA-amplicon or no amplicon with increasing amount of SYBR- or Eva- Green. The resulting melting curves and melting peaks are represented. Yellow, orange and red melting curves and peaks: SIV 3.2-kb/HIV 2.9-kb full env amplicon melting profile with 1, 2 and 4 μl dye respectively. Light, medium and dark blue melting curves and peaks: SIV 950-bp/HIV 650-bp MS RNA amplicon melting profile with 1, 2 and 4 μl dye respectively. Light grey, dark grey and black melting curves/peaks: negative reaction melting profile for 1, 2 and 4 μl dye respectively.
(TIF)

S4 Fig Analysis of a 96-well plate HIV SGA EvaGreen melting profile using the “Graph-2steps” method. From the full plate HIV SGA EvaGreen melting curve profile (1), the curves with higher intensity level corresponding to the presence of an amplification product are selected (step 1). From the corresponding melting peak profile (2), the double peaks with Tm of 80 and 84°C (red curves) specific for the 2.9-kb full env amplicon are selected (step 2). The corresponding positions on the plate map (3) correspond to the wells containing the full env amplicon. This 2 steps graphical analysis method of HIV SGA melting profile can be used with EvaGreen dye only and will require between 3 and 4 minutes for a 96-well plate.
(TIF)

S5 Fig Comparison between gel electrophoresis and EvaGreen melting profiles of 96 HIV SGA PCR reactions. HIV SGA was performed using cDNA samples from HIV infected Jurkat cells at a working dilution of 10^5^ fold. 1.2 μl of each 96 SGA reactions were used to carry out both agarose gel and EVA green melting analysis. The profiles corresponding to the 23 full env (3.2-kb), 5 MS RNA (650-bp), 6 intermediate size and 7 small size amplicon-containing reactions are indicated using red, blue, orange and green arrowheads and curves respectively. Melting profiles are represented as a full plate view (top panels), or partial to highlight differences between profiles (bottom panels).
(TIF)

S6 Fig SIV SGA EvaGreen melting profile analysis using the “Excel sheet/values” method. The results (Tm) and Raw data (fluorescence intensity values) corresponding to the EvaGreen melting profile (left panel) of a full SIV SGA 96-well plate are extracted as an excel document and used to prepare a working sheet (center panel) as follows: out of the 95 reading time points, choose one in the thirty first (here reading 10) and select the corresponding reading values for EvaGreen reporter fluorescence (here filter 1) and ROX passive reference (here filter 4) for the full plate. Add a column with the ratio EVA green / ROX values (here F1/F4), corresponding to a reasonable approximation of the plotted values. From the results data sheet, import the 2 columns corresponding to the two first peaks detected by the software (here Tm1 and Tm2). Filter the data present in the EvaGreen/ROX ratio column for values more than 0.4 (corresponding to the wells containing a PCR product) and in the Tm1 column for values less than 83°C (corresponding to the full env specific low Tm peak). The wells selected are the one containing the full env 3.2-kb amplicon. This method can be used with EvaGreen melting only and for SIV SGA only with most of the software and will require between 5 and 10 minutes for a 96well plate, most of the time needed for the elaboration of the working sheet. However, considering that the filtering step is lasting few second only, this method is well adapted for the analysis of larger scale data (384-well format for example) that should also require around 5 to 10 minutes.
(TIF)

S7 Fig SIV SGA EvaGreen melting profile analysis using the “Graph-1click” method. A full plate SIV SGA EvaGreen melt profiling was performed on a CFX96 Touch Real-Time PCR detection system instrument (Bio-Rad) and the onboard CFX manager software was used to apply the “Graph-1 click” method as follows: from the full plate melting peak profile (A), select by clicking the curves with a peak at lower Tm (B, top right) and choose “selected wells—view only”; the wells with full env amplicon are selected (C). This method can be used with EvaGreen melting for both HIV and SIV SGA and SYBR Green for SIV only (2 clicks instead of 1) and will require between 10 and 30 seconds for a 96-well plate. However, this method requires the possibility to “click and select” the curves which is not present on every QPCR software.
(TIF)

S8 Fig 384-well format SIV env SYBR Green and EvaGreen LAMP. 1.2 μl of 96 SIV env SGA nested reactions were transferred to a 384-well plate containing the melting mix and the melting run was performed on a CFX384 Touch Real time PCR detection system instrument (Bio-Rad). The melting curve and melting peak profiles obtained with SYBR Green (left) and EvaGreen (right) showed differences in fluorescence intensity and/or peak shape between negative reactions (light green curves) and env (red curves) and spliced RNA (blue curves) amplicon containing reactions. Analysis of the melting profile using the fast “Graph-1click” method highlighted the wells containing the 3.2-kb env amplicon on the plate (bottom).
(TIF)

S1 Table Timing for LAMP screening of 96- and 384-well SIV/HIV SGA PCR plates. (DOCX)
