# Supplementary figures and images for: A New Method for Rapid Screening of End-Point PCR Products: Application to Single Genome Amplified HIV and SIV Envelope Amplicons
Source: PLoS One. 2015 Jun 8;10(6):e0128188. doi: 10.1371/journal.pone.0128188 (PMC4460086; doi:10.1371/journal.pone.0128188)

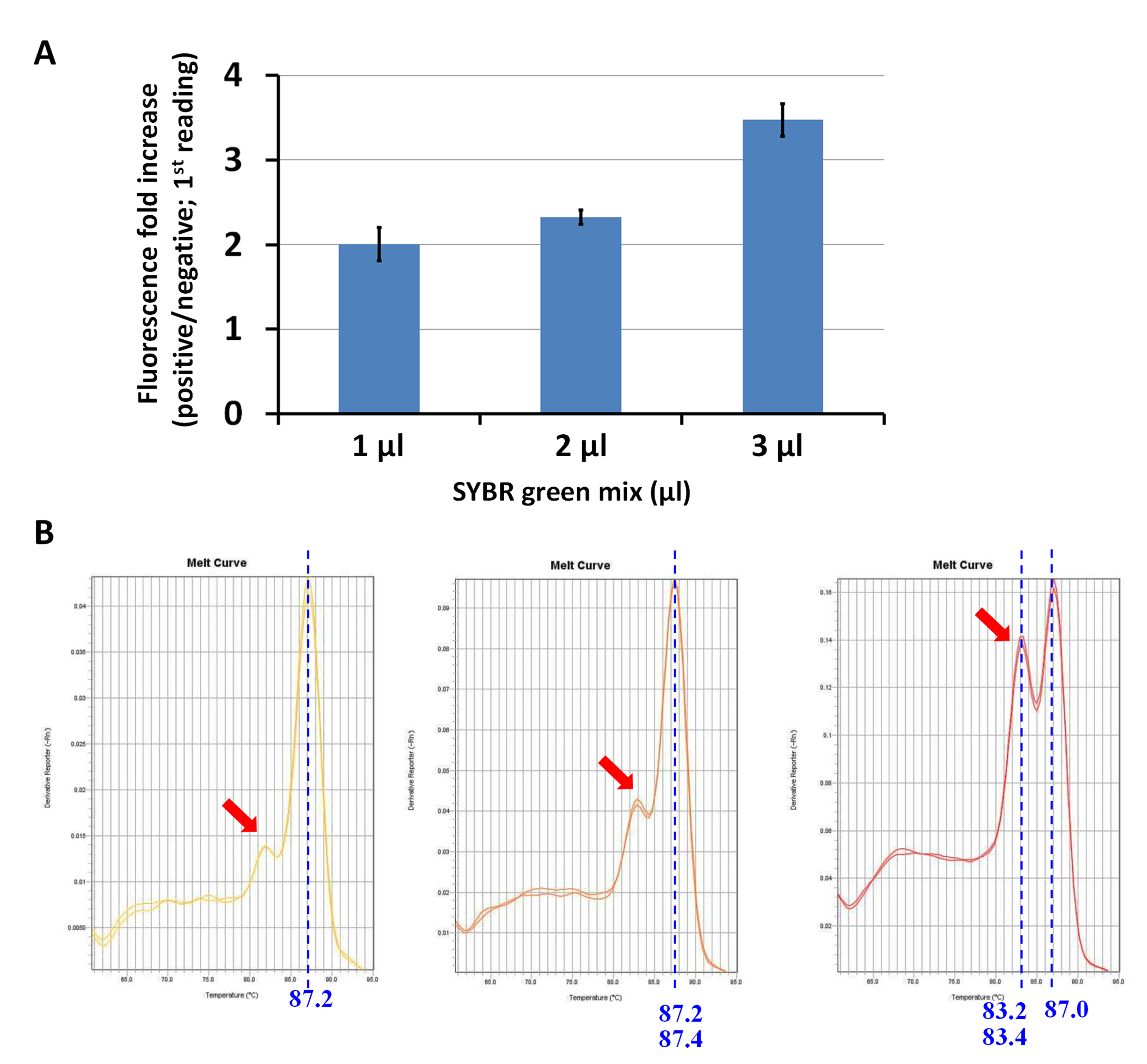

Supplement: S1 Fig — (A) Effect of increasing amount of SYBR Green on relative fluorescence intensity between positive and negative reactions. The raw data were extracted from the melting run presented in Fig 1 and used to calculate the corrected initial fluorescence intensity values (SYBR Green signal intensity/ROX signal intensity for each sample, using the intensity values from the first reading) corresponding to the best approximation of the plotted values. The ratio between the values corresponding to positive and negative reactions was calculated for each dose of SYBR Green and expressed as a fold increase in fluorescence intensity. (B) Effect of increasing amount of SYBR Green on melting peak profile of the 3.2-kb SIV env SGA-amplicon. The melting peaks obtained for 1.2 μl SGA PCR reaction melted with 1 μl (left panel), 2 μl (middle panel) and 4 μl (right panel) SYBR Green are represented separately to highlight the relative height between the major and minor (red arrow) peaks. The peaks detected by the onboard software are indicated with a blue dashed line and the corresponding Tm values are given in blue. (TIF) [file pone.0128188.s001.tif]

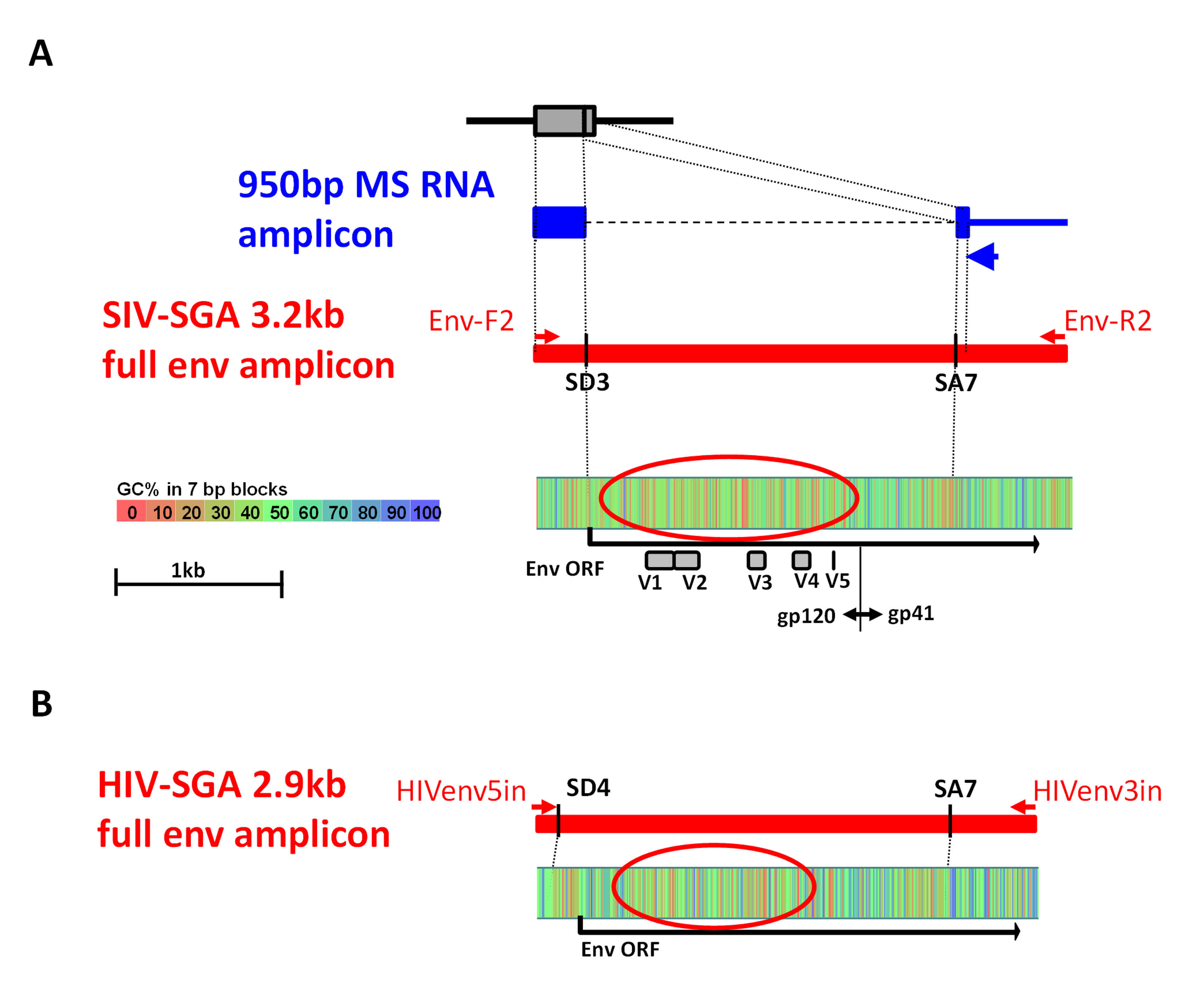

Supplement: S2 Fig — (A) SIV SGA full env and MS RNA amplicons. The %GC (heat map), Env protein ORF (dark arrow), V1 to V5 variable regions (grey boxes) and gp120/gp41 junction (double headed arrow) present in the 3.2-kb full env amplicon (red line, and red arrows for primers) are shown. The blue boxes, corresponding to the region of the 950-bp amplicon sequenced using the primer indicated as a blue arrowhead, are represented above their corresponding part in the full env amplicon and correspond to a junction between the donor splicing site SD3 and the acceptor splicing site SA7. A similar junction was observed in the sequence called “SIV tat and rev genes, complete cds” corresponding to one of the SIV MS RNA and represented as a grey box. The oval delimited with a red line corresponds to the GC poor area present in the SD3-SA7 intron and most likely responsible for the shoulder on low temperature side specific for the full env amplicon. (B) HIV SGA full env amplicon. Similar to SIV, HIV amplicon show a GC poor region (red oval) located in the SD4-SA7 intron. (TIF) [file pone.0128188.s002.tif]

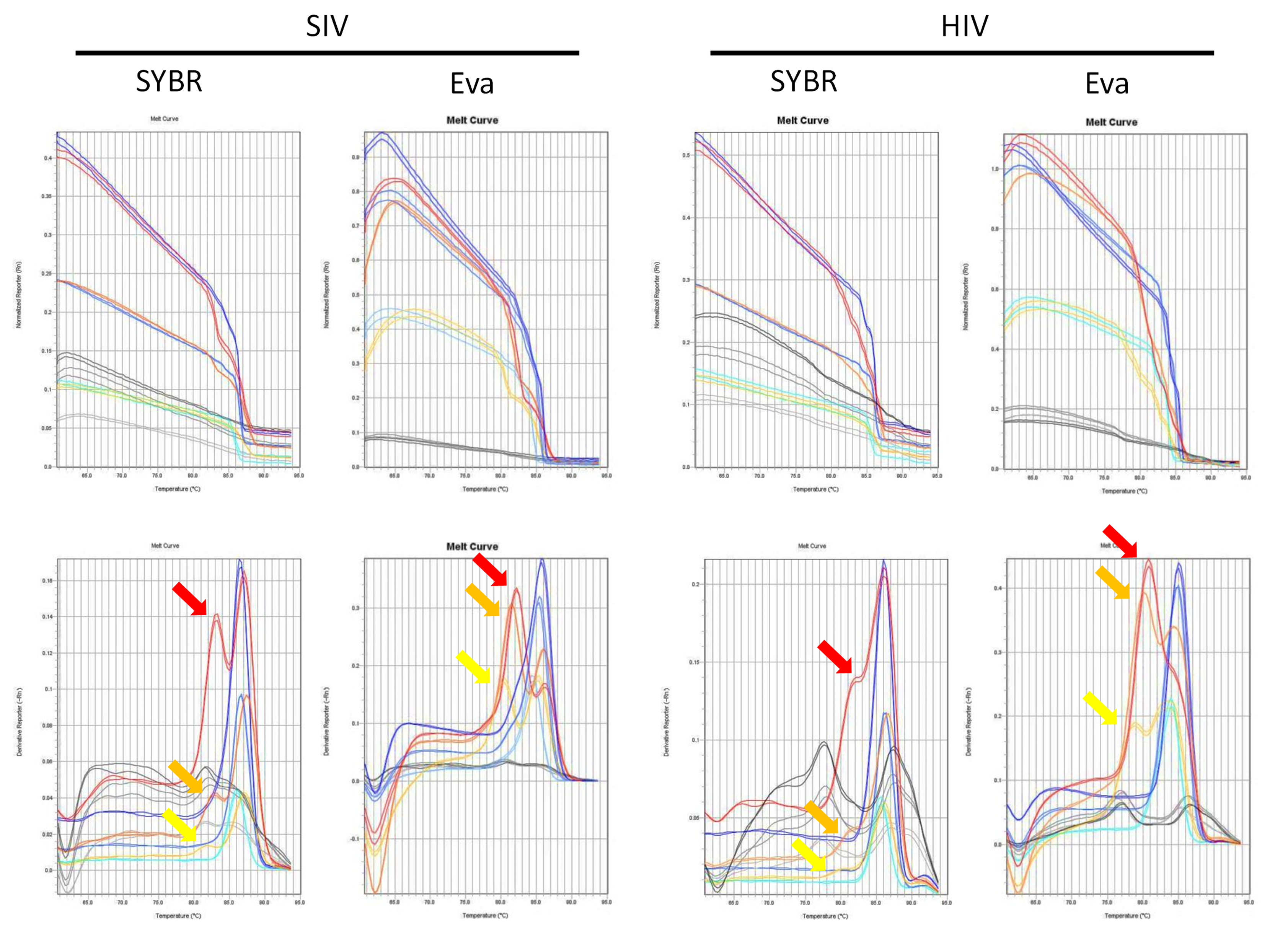

Supplement: S3 Fig — Melting reactions were performed using 1.2 μl of a SGA PCR reaction containing the SIV or HIV env or MS RNA SGA-amplicon or no amplicon with increasing amount of SYBR- or Eva- Green. The resulting melting curves and melting peaks are represented. Yellow, orange and red melting curves and peaks: SIV 3.2-kb/HIV 2.9-kb full env amplicon melting profile with 1, 2 and 4 μl dye respectively. Light, medium and dark blue melting curves and peaks: SIV 950-bp/HIV 650-bp MS RNA amplicon melting profile with 1, 2 and 4 μl dye respectively. Light grey, dark grey and black melting curves/peaks: negative reaction melting profile for 1, 2 and 4 μl dye respectively. (TIF) [file pone.0128188.s003.tif]

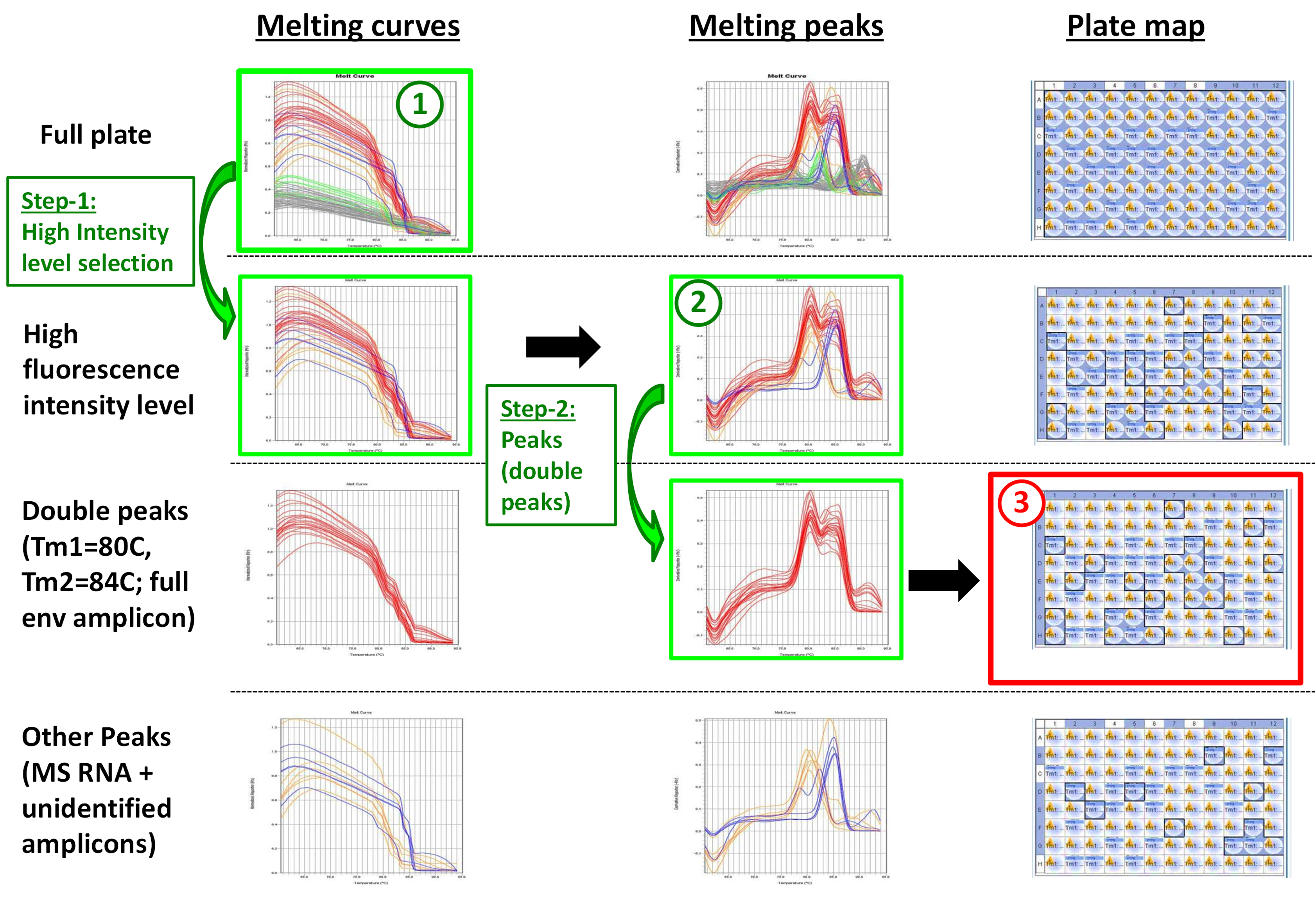

Supplement: S4 Fig — From the full plate HIV SGA EvaGreen melting curve profile (1), the curves with higher intensity level corresponding to the presence of an amplification product are selected (step 1). From the corresponding melting peak profile (2), the double peaks with Tm of 80 and 84°C (red curves) specific for the 2.9-kb full env amplicon are selected (step 2). The corresponding positions on the plate map (3) correspond to the wells containing the full env amplicon. This 2 steps graphical analysis method of HIV SGA melting profile can be used with EvaGreen dye only and will require between 3 and 4 minutes for a 96-well plate. (TIF) [file pone.0128188.s004.tif]

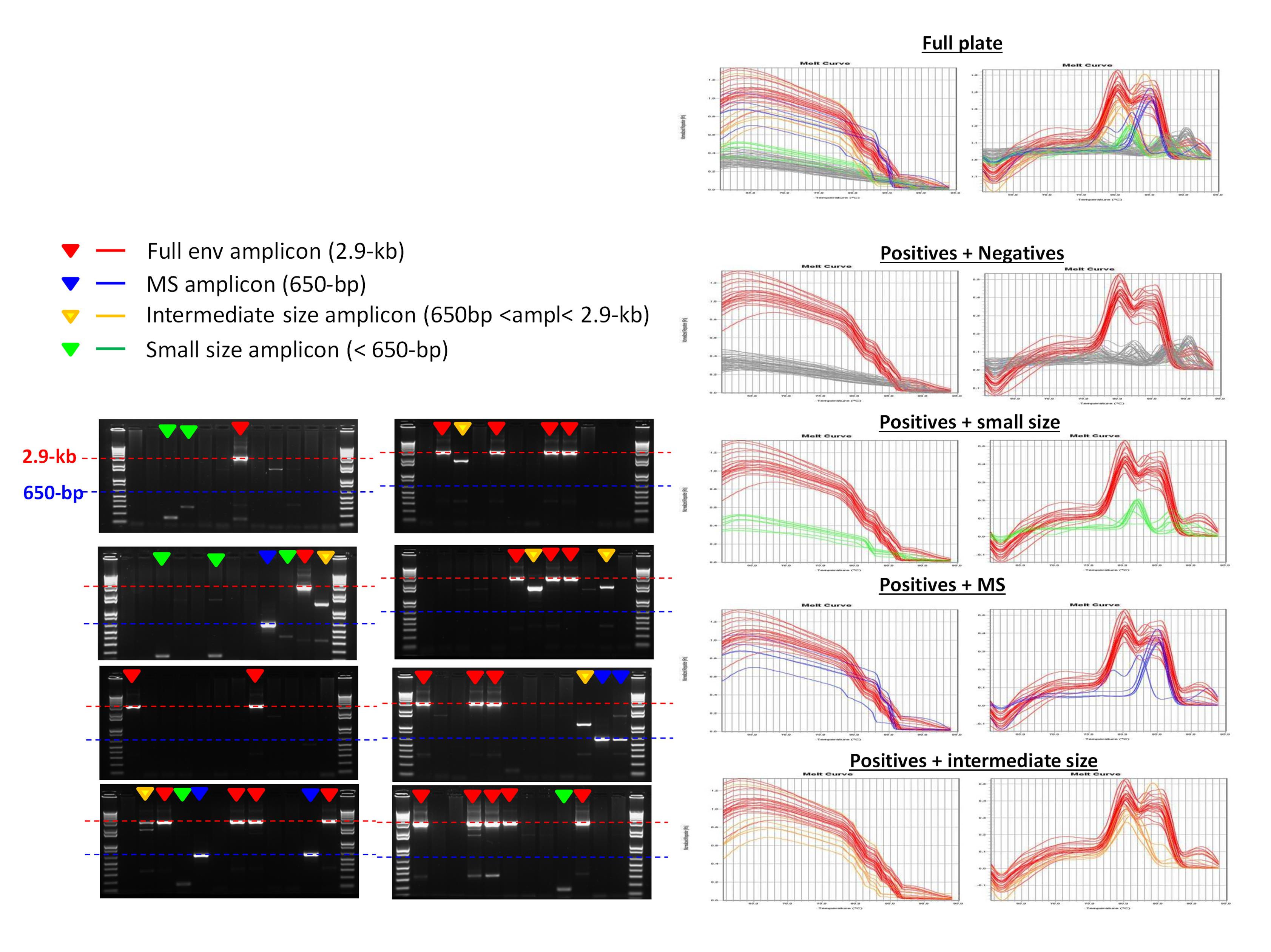

Supplement: S5 Fig — HIV SGA was performed using cDNA samples from HIV infected Jurkat cells at a working dilution of 105 fold. 1.2 μl of each 96 SGA reactions were used to carry out both agarose gel and EVA green melting analysis. The profiles corresponding to the 23 full env (3.2-kb), 5 MS RNA (650-bp), 6 intermediate size and 7 small size amplicon-containing reactions are indicated using red, blue, orange and green arrowheads and curves respectively. Melting profiles are represented as a full plate view (top panels), or partial to highlight differences between profiles (bottom panels). (TIF) [file pone.0128188.s005.tif]

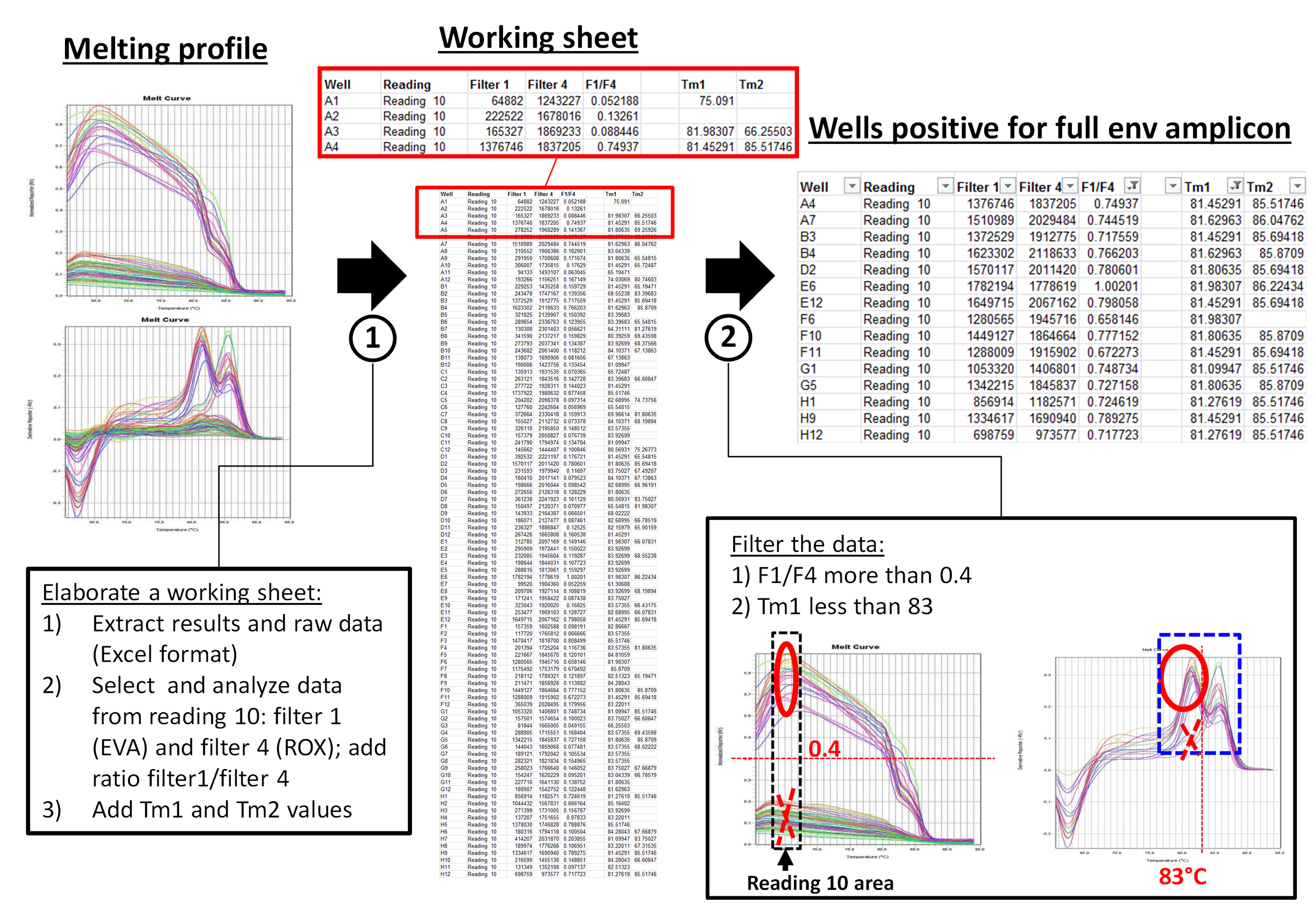

Supplement: S6 Fig — The results (Tm) and Raw data (fluorescence intensity values) corresponding to the EvaGreen melting profile (left panel) of a full SIV SGA 96-well plate are extracted as an excel document and used to prepare a working sheet (center panel) as follows: out of the 95 reading time points, choose one in the thirty first (here reading 10) and select the corresponding reading values for EvaGreen reporter fluorescence (here filter 1) and ROX passive reference (here filter 4) for the full plate. Add a column with the ratio EVA green / ROX values (here F1/F4), corresponding to a reasonable approximation of the plotted values. From the results data sheet, import the 2 columns corresponding to the two first peaks detected by the software (here Tm1 and Tm2). Filter the data present in the EvaGreen/ROX ratio column for values more than 0.4 (corresponding to the wells containing a PCR product) and in the Tm1 column for values less than 83°C (corresponding to the full env specific low Tm peak). The wells selected are the one containing the full env 3.2-kb amplicon. This method can be used with EvaGreen melting only and for SIV SGA only with most of the software and will require between 5 and 10 minutes for a 96well plate, most of the time needed for the elaboration of the working sheet. However, considering that the filtering step is lasting few second only, this method is well adapted for the analysis of larger scale data (384-well format for example) that should also require around 5 to 10 minutes. (TIF) [file pone.0128188.s006.tif]

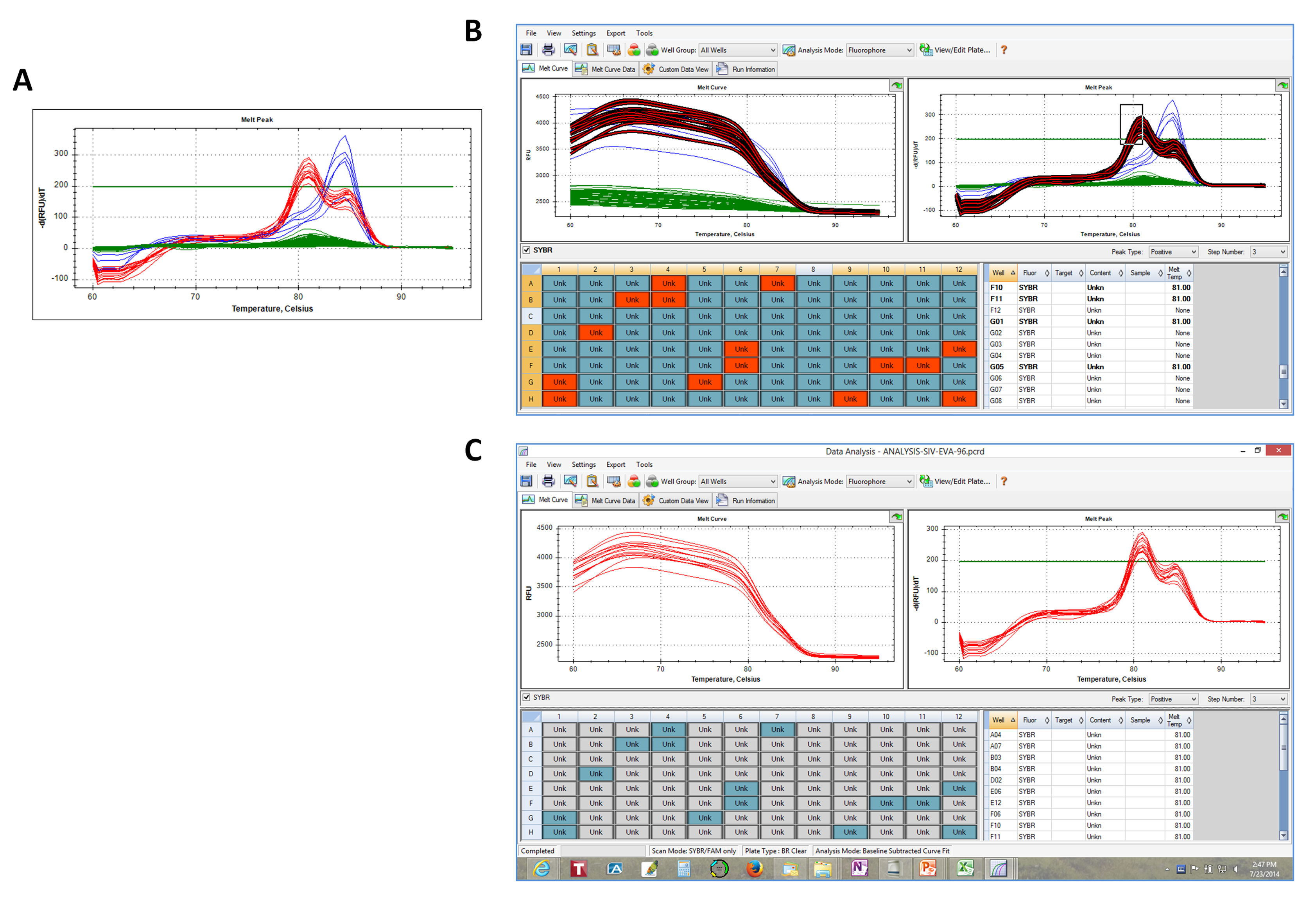

Supplement: S7 Fig — A full plate SIV SGA EvaGreen melt profiling was performed on a CFX96 Touch Real-Time PCR detection system instrument (Bio-Rad) and the onboard CFX manager software was used to apply the “Graph-1 click” method as follows: from the full plate melting peak profile (A), select by clicking the curves with a peak at lower Tm (B, top right) and choose “selected wells—view only”; the wells with full env amplicon are selected (C). This method can be used with EvaGreen melting for both HIV and SIV SGA and SYBR Green for SIV only (2 clicks instead of 1) and will require between 10 and 30 seconds for a 96-well plate. However, this method requires the possibility to “click and select” the curves which is not present on every QPCR software. (TIF) [file pone.0128188.s007.tif]

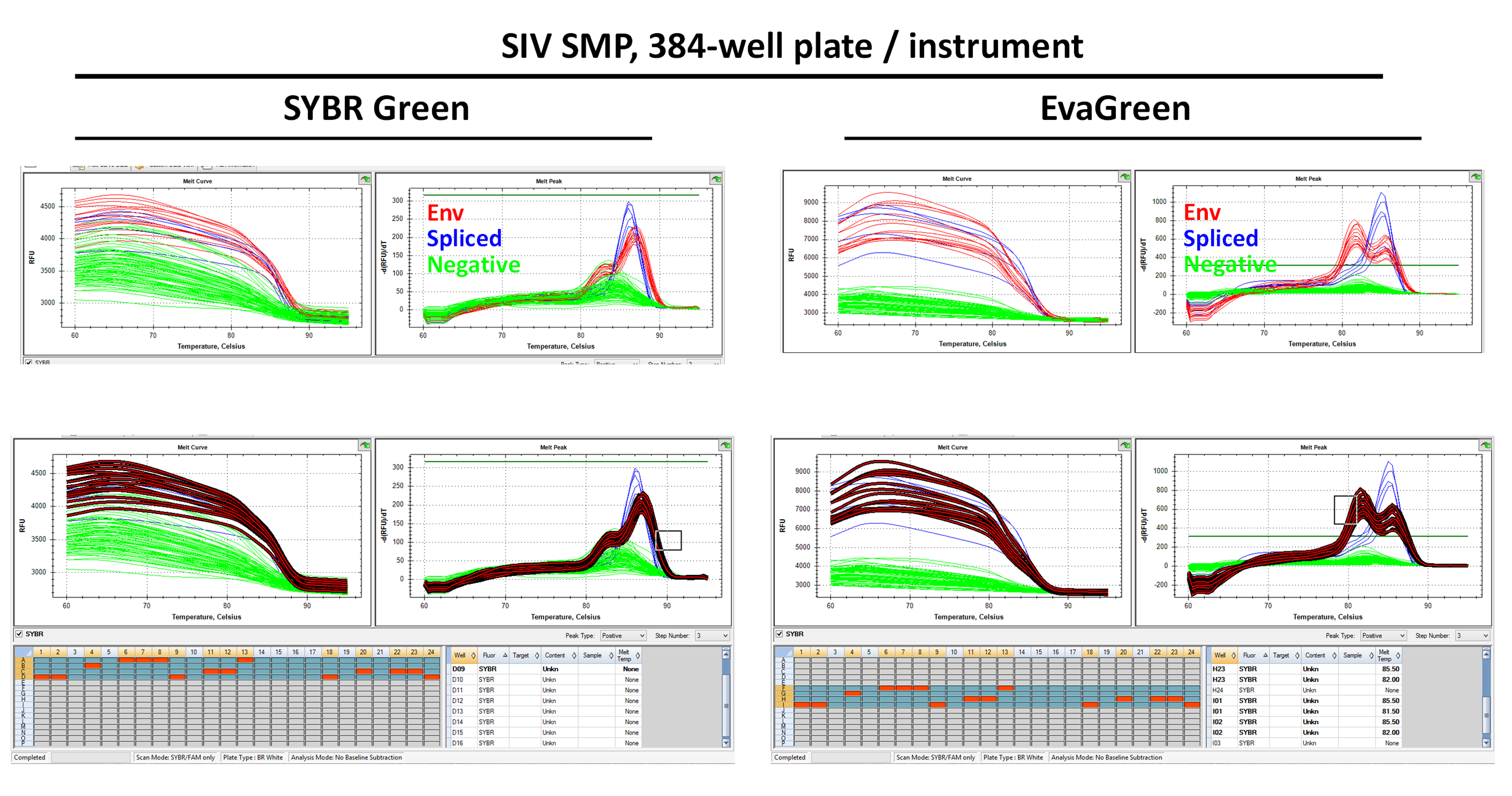

Supplement: S8 Fig — 1.2 μl of 96 SIV env SGA nested reactions were transferred to a 384-well plate containing the melting mix and the melting run was performed on a CFX384 Touch Real time PCR detection system instrument (Bio-Rad). The melting curve and melting peak profiles obtained with SYBR Green (left) and EvaGreen (right) showed differences in fluorescence intensity and/or peak shape between negative reactions (light green curves) and env (red curves) and spliced RNA (blue curves) amplicon containing reactions. Analysis of the melting profile using the fast “Graph-1click” method highlighted the wells containing the 3.2-kb env amplicon on the plate (bottom). (TIF) [file pone.0128188.s008.tif]
