# Supplementary material for: A New Method for Rapid Screening of End-Point PCR Products: Application to Single Genome Amplified HIV and SIV Envelope Amplicons
Source: PLoS One. 2015 Jun 8;10(6):e0128188. doi: 10.1371/journal.pone.0128188 (PMC4460086; doi:10.1371/journal.pone.0128188)
Supplement: S1 Table — (DOCX) [file pone.0128188.s009.docx]

**S1 Table. Timing for LAMP screening of 96- and 384-well SIV/HIV SGA PCR plates**

| **LAMP step** | **96-well format** | **384-well format** |
| --- | --- | --- |
| Melting Plate setup | 10 min. | 20 min. |
| Melting run | 25 to 40 min. | 35 min.* |
| Melting profile analysis** | 10 sec. to 8 min. | 10 sec. to 16 min. |
| Total time | 35 to 58 min. | 55 to 71 min. |

* Correspond to the protocol used with the CFX384 Touch (Bio-Rad)

** The timing is different depending on the analysis method used (see Table 2 and text for detail)
